# Frequency of Tongue Cleaning Impacts the Human Tongue Microbiome Composition and Enterosalivary Circulation of Nitrate

**DOI:** 10.3389/fcimb.2019.00039

**Published:** 2019-03-01

**Authors:** Gena D. Tribble, Nikola Angelov, Robin Weltman, Bing-Yan Wang, Sridhar V. Eswaran, Isabel C. Gay, Kavitha Parthasarathy, Doan-Hieu V. Dao, Katherine N. Richardson, Nadia M. Ismail, Iraida G. Sharina, Embriette R. Hyde, Nadim J. Ajami, Joseph F. Petrosino, Nathan S. Bryan

**Affiliations:** ^1^Department of Periodontics, School of Dentistry, The University of Texas Health Science Center Houston, Houston, TX, United States; ^2^Division of Cardiology, Department of Internal Medicine, McGovern Medical School, The University of Texas Health Science Center Houston, Houston, TX, United States; ^3^SynBioBeta, Pleasant Hill, CA, United States; ^4^Alkek Center for Metagenomics and Microbiome Research, Baylor College of Medicine, Houston, TX, United States; ^5^Department of Molecular Virology and Microbiology, Baylor College of Medicine Houston, TX, United States; ^6^Department of Molecular and Human Genetics, Baylor College of Medicine, Houston, TX, United States

**Keywords:** oral microbiome, microbiome, microbial ecology, host-microbial symbiosis, nitrate, nitric oxide

## Abstract

The oral microbiome has the potential to provide an important symbiotic function in human blood pressure physiology by contributing to the generation of nitric oxide (NO), an essential cardiovascular signaling molecule. NO is produced by the human body via conversion of arginine to NO by endogenous nitric oxide synthase (eNOS) but eNOS activity varies by subject. Oral microbial communities are proposed to supplement host NO production by reducing dietary nitrate to nitrite via bacterial nitrate reductases. Unreduced dietary nitrate is delivered to the oral cavity in saliva, a physiological process termed the enterosalivary circulation of nitrate. Previous studies demonstrated that disruption of enterosalivary circulation via use of oral antiseptics resulted in increases in systolic blood pressure. These previous studies did not include detailed information on the oral health of enrolled subjects. Using 16S rRNA gene sequencing and analysis, we determined whether introduction of chlorhexidine antiseptic mouthwash for 1 week was associated with changes in tongue bacterial communities and resting systolic blood pressure in healthy normotensive individuals with documented oral hygiene behaviors and free of oral disease. Tongue cleaning frequency was a predictor of chlorhexidine-induced changes in systolic blood pressure and tongue microbiome composition. Twice-daily chlorhexidine usage was associated with a significant increase in systolic blood pressure after 1 week of use and recovery from use resulted in an enrichment in nitrate-reducing bacteria on the tongue. Individuals with relatively high levels of bacterial nitrite reductases had lower resting systolic blood pressure. These results further support the concept of a symbiotic oral microbiome contributing to human health via the enterosalivary nitrate-nitrite-NO pathway. These data suggest that management of the tongue microbiome by regular cleaning together with adequate dietary intake of nitrate provide an opportunity for the improvement of resting systolic blood pressure.

## Introduction

**Figure 1:**
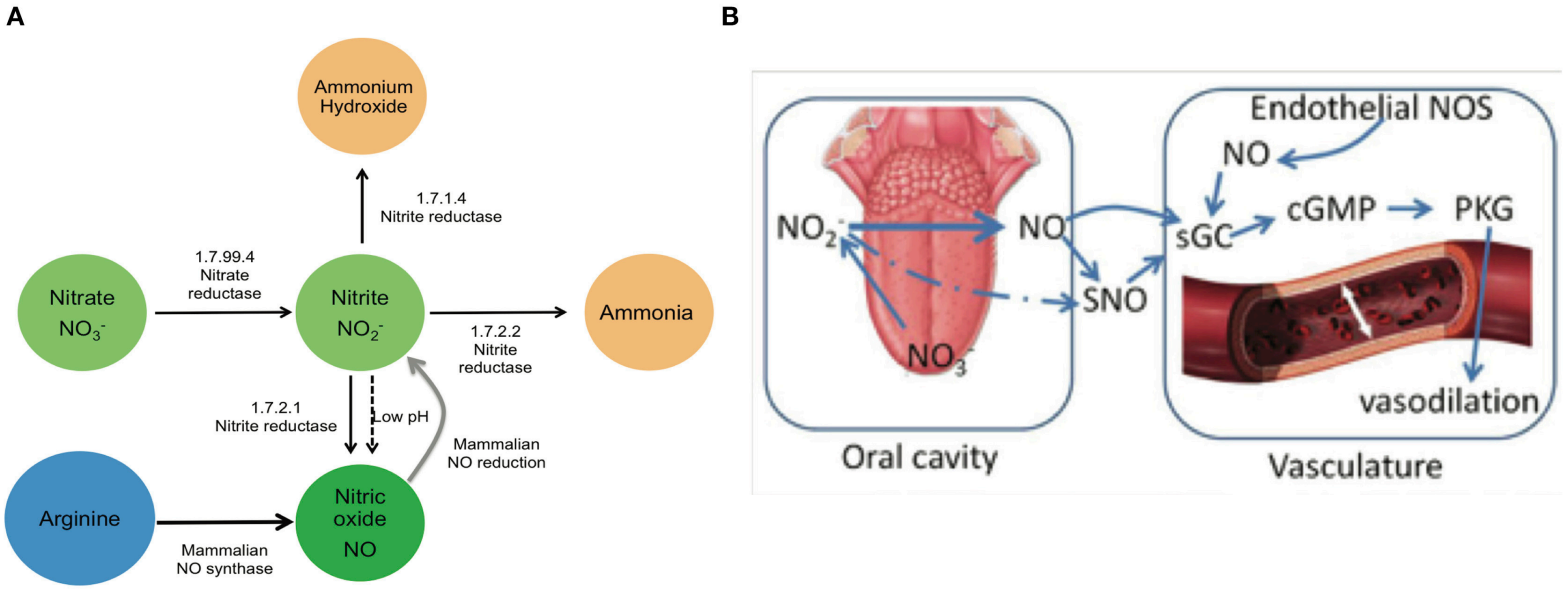
Enterosalivary circulation of nitrate. **(A)** Bacterial and mammalian enzymes in nitrate metabolism. The bio-activation of nitrate from dietary or endogenous sources requires its initial reduction to nitrite, and because mammals lack nitrate reductase enzymes, this conversion is mainly carried out by commensal bacteria. The presence of bacteria encoding nitrate reductase (1.7.99.4) is predicted to be critical for enterosalivary circulation. Nitrite represents a major storage form of NO in blood and tissues: Nitrite- NO conversions can be driven by enzymatic and chemical mechanisms. 1.7.99.4—Nitrate reductase, 1.7.2.1—Nitrite reductase (NO-forming), 1.7.2.2—Nitrite reductase (cytochrome; ammonia-forming), 1.7.1.4—Nitrite reductase (NAD(P)H). **(B)** Interactions of oral nitrate metabolites with the host vasculature. Dietary nitrate converted to nitrite in the oral cavity may be converted to NO and/or react with endothelial and plasma proteins to form S-nitrosothiol (SNO). Nitrite along with NO and SNO has been shown to activate soluble guanylyl cyclase (sGC) and increase cGMP levels in tissue. Nitrite-NO-SNO, whether from dietary sources or endothelial production, improves vascular tone via cell signaling through cGMP/PKG and stimulation of smooth muscle relaxation.

The human oral cavity is an important habitat for microbes, and a healthy mouth can harbor upwards of ten billion bacteria (Loesche, 1993). Cardiovascular research has identified a potential role for the oral microbiome in human health via the conversion of dietary nitrate into nitrite, the bioactive storage pool available for spontaneous conversion to nitric oxide (NO) (Lundberg and Govoni, 2004; Lundberg et al., 2008). Continuous generation of NO is essential for the integrity of the cardiovascular system, and decreased production or bioavailability of NO is central to the development of many heart-related disorders (Lundberg et al., 2004, 2009; Bryan and Loscalzo, 2017). The human body is able to produce NO directly by the five-electron oxidation of L-arginine (Moncada and Higgs, 1993) by endothelial nitric oxide synthases (eNOS; Figure 1A). However, the eNOS gene is polymorphic, and the pathway can become dysfunctional with age (Niu and Qi, 2011). Failure to produce sufficient NO is causal for the onset and progression of a number of cardiovascular diseases, including hypertension and atherosclerosis (Taddei et al., 2001; Torregrossa et al., 2011). The enterosalivary nitrate-nitrite-NO pathway in humans appears to serve as an alternative pathway for production of bioactive NO, supplementing host endothelial NO production. This pathway functions via the bacterial conversion of dietary nitrate into nitrites that can then be converted to NO and participate in the regulation of endothelial vasodilation (Figure 1B) (Benjamin et al., 1994; Lundberg et al., 1994). This diet-dependent pathway relies upon commensal oral bacteria located on the tongue dorsum to perform the first step (nitrate reduction to nitrite) since mammals lack a functional nitrate reductase (Lundberg et al., 2004; Doel et al., 2005).

Although there is compelling evidence supporting the role of the tongue microbiome in the enterosalivary nitrate-nitrite-NO pathway, this remains a relatively unexplored area of human-microbial mutualism and many important questions must be addressed. Previous studies have demonstrated that nitrite-induced reductions in blood pressure are inhibited by antiseptic chlorhexidine (CHX) or other antibacterial mouthwashes (Tannenbaum et al., 1976; Govoni et al., 2008; Petersson et al., 2009; Kapil et al., 2013; McDonagh et al., 2015; Woessner et al., 2016; Mitsui and Harasawa, 2017). The focus of these studies has been from the perspective of cardiovascular physiology, and little information is available regarding the oral health status or habits of subjects in these studies. In this study we approach this symbiotic relationship from the perspective of oral health using a cohort of 27 oral health professionals with both excellent cardiovascular and oral health. These subjects used a CHX mouthwash for 7 days, and we assessed the response of resting systolic blood pressure and tongue microbiome community composition before and after exposure to this potent antimicrobial agent. The human oral microbiome is highly variable between individuals; therefore, we hypothesized that there would be inter-subject variability in response to CHX dependent upon the composition of the baseline tongue microbiota. In this study we provide the first assessment of the tongue microbiome in parallel with resting blood pressure in healthy individuals treated with CHX.

## Materials and Methods

### Subject Recruitment

Subjects were recruited from the faculty, staff, dental and dental hygiene students of the University of Texas Health Science Center at Houston School of Dentistry. For inclusion in the study, subjects were over the age of 18 and capable of giving consent, were non-smokers, had not used antibiotics within the previous 3 months, had no history of bone loss, and no history of hypertension. Volunteers were evaluated for oral health, including the use of a standard periodontal exam, with spot probing for bleeding and loss of attachment, and an oral health subject history. During assessment, subjects were excluded upon discovery of bleeding on probing at more than 10% of sites, <24 teeth, attachment loss of more than 4 mm at any site, the presence of oral hard or soft tissue lesions, or a resting blood pressure of >130 mm/Hg. Thirty four subjects were screened, and 6 subjects excluded after oral exam and initial blood pressure measurements (Supplemental Data Figure 1). Of the 28 subjects initiating the study, one was discontinued due to antibiotic usage at the first follow-up visit, and one (subject NiOx38) was discontinued at the final visit. Data from the first three time points for NiOx38 were used for data analysis. Twenty-six subjects completed the final visit of the study. The study protocol was approved by the Committee for the Protection of Human Subjects at the University of Texas Health Science Center at Houston (HSC-DB-14-0078). Subject demographic data is shown in Table 1.

**Table 1:** Demographics.

	**Count**	**Percent**
**GENDER**		
Male	10	37%
Female	17	63%
**AGE**		
20–30	16	59%
30–40	6	22%
40–50	3	11%
50 and over	2	8%
**RACE AND ETHNICITY**		
African-American	3	10%
Caucasian	11	40%
Asian	5	17%
Hispanic	6	21%
Middle Eastern	1	3%
Asian-Caucasian	1	3%
**EDUCATION**		
High School Diploma	1	4%
Some college	5	19%
Associates Degree	1	4%
Bachelor's degree	11	41%
Professional degree	8	30%
No response	1	4%

*27 subjects were enrolled in the study, and distribution of subjects by major demographic measures are shown*.

### Study Design And Procedures

The study uses a repeated measures linear mixed model to detect changes in systolic blood pressure over 14 days. Power analysis estimated that 24 subjects needed to complete the study in order to detect a medium effect size at 80% power. The study uses longitudinal repeated measures of resting systolic blood pressure as the primary outcome. The study protocol consisted of 4 visits: day 1 (baseline), day 7 (after 7 days CHX treatment), day 10 (3 days recovery) and day 14 (7 days recovery). Study visits were always scheduled at noon, and lasted for 1 h to allow repeated resting blood pressure measurements. For the duration of each visit, subjects were seated in a dental chair in the upright position, with ankles uncrossed. At the first visit, the subjects completed general demographics and medication questionnaires, and an oral hygiene survey.

At each visit blood pressure measurements were taken three times with an interval of 15 min between the measurements. The first blood pressure reading was taken after at least 10 min seated in the chair (Kallioinen et al., 2017). Blood pressure was taken on alternating arms at each time point, beginning with the right arm. Tongue scrapings and saliva were collected at each visit. Unstimulated whole saliva was taken using the passive drool method into a cryovial with a saliva collection aid (Salimetrics, LLC). Tongue samples were collected by drawing a sterile stainless steel tongue scraper with gentle pressure across the tongue dorsum from the posterior to the mid-anterior. 50 ul of tongue sample was transferred to MoBio PowerSoil tubes for bacterial DNA extraction and 16s rRNA community analysis. The remainder of the sample was transferred to cryovials containing freezing medium [(0.045% K_2_HPO_4_, 0.045% KH_2_PO_4_), 0.09% NaCl, 0.09% (NH_4_)_2_SO_4_, 0.018% MgSO_4_, 0.038% EDTA, 0.04% Na_2_CO_3_, 0.02% dithiothreitol, 0.2% Bacto-agar, 5% glycerol] and stored at −80°C for preservation of viable bacteria. At each visit, after collection of oral samples, subjects performed a nitrate rinse for 2 min with 50 ml of a solution of 1 mM sodium nitrate to assess *in vivo* nitrate reduction by oral bacteria. After 2 min, subjects spit the 50 ml nitrate rinse sample into a collection tube. Samples were frozen at −80°C, and subsequently analyzed for nitrate/nitrite content as described in Nitrate-Nitrite Quantification in Subject Samples.

The treatment in this study was use of 0.12% Chlorhexidine gluconate mouthwash (Peridex) twice a day for 7 days. At the completion of the first visit each subject was provided with a prescription oral mouthwash with 0.12% Chlorhexidine gluconate (CHX) and instructed to rinse with ½ ounce for 30 s twice a day as part of their normal oral hygiene in the morning and evening. Subjects were asked to discontinue use of any other mouthwash for the first 7 days, but otherwise maintain their normal oral care regimen, as documented by the oral hygiene survey (Table 2). At their second visit (day 7 time point) subjects were instructed to cease use of CHX and return to their normal oral health care products. Subjects returned at day 10 and 14 for blood pressure and oral sampling during the recovery from treatment phase.

**Table 2:** Oral hygiene survey.

	**Count**	**Percent**
**BRUSH TEETH**		
Twice a day	22	81%
Three times a day	5	19%
**FLOSS**		
Several times per month	1	4%
Several times per week	8	30%
Once a day	16	67%
Twice a day	2	7%
**CLEAN TONGUE**		
Less than once a week	3	12%
Weekly	1	3%
Once a day	13	48%
Twice a day or more	10	37%
**MOUTHWASH USE**		
As needed	5	19%
Once a day	16	59%
Twice a day	6	22%
**MOUTHWASH INGREDIENT**		
Essential oils	11	41%
Cetylpyridinium chloride	10	37%
No response	6	22%
**TYPE OF TOOTHBRUSH**		
Manual	10	37%
Electric	12	44%
Both manual and electric	5	19%
**VISITS TO DENTIST PER YEAR**		
None	1	4%
Once	12	44%
Twice or more	14	52%

*Subject responses to the oral hygiene survey are summarized*.

### Bacterial Viable Counts and Nitrate Reduction Assay

A subset of six subjects performed an 8-h time course to assess bacterial response to CHX. Tongue samples were obtained from the dorsum using a stainless steel tongue scraper hourly for 8 h after rinsing with CHX. A total of 10 samples were collected, at baseline, after CHX rinse, and hourly afterward for 8 h. Bacterial samples were collected from the posterior of the tongue dorsum by gently stroking the surface with a 0.6 cm diameter metal scraper three times, over a ~0.6 cm^2^ surface area.

A different section of the tongue was sampled at each time point. Bacterial sample was transferred to a cryovial containing freezing medium and stored immediately at −80°C for preservation of viable bacteria. For calculation of viable counts, samples were thawed on ice and serially diluted five times at 20-fold increments. The final three dilutions were dropped in 5 ul aliquots in triplicate onto Trypticase Soy Blood agar supplemented with 0.1%Yeast/7.5 uM hemin/3 uM vitamin K (TSBY-S) and incubated anaerobically at 37°C for 72 h. For calculation of *ex-vivo* nitrate reducing activity in tongue samples, samples were standardized to the same starting OD660 in sterile saline, and 100 ul aliquots were grown in 24 well tissue culture plates with 1 ml of TSBY-S broth supplemented with 5 mM nitrate solution. The plates were incubated for 24 h anaerobically and statically at 37°C. Biofilm supernatants were collected and processed for nitrite-nitrate measurements using the Nitrate reduction test (Sigma-Aldrich) as recommended.

### Blood Pressure Statistical Analysis

Blood pressure data was analyzed using repeated measures two-way ANOVA, for both systolic and diastolic readings. The first factor was time point, and the second factor was subject. Additional analysis was done with the second factor as gender, race-ethnicity, and other demographic variables. *Post-hoc* tests between time points and within individuals were done with Bonferroni analysis and Fishers Least Significant Difference. Association of blood pressure changes with subject metadata was assessed by Spearman's correlation. Differences between population distributions were assessed by the F-test for Variances. Power analysis for study design was done with G^*^Power. All other statistical analysis and graphing were performed with StatPlus for Excel (Microsoft).

### Nitrate-Nitrite Quantification in Subject Samples

Nitrate and nitrite were determined by using HPLC, as described previously (Rassaf et al., 2002; Bryan and Grisham, 2007; Jiang et al., 2012b). This method employs ion chromatography with on-line reduction of nitrate to nitrite and subsequent post-column derivatization with the Griess reagent (ENO-20, EiCom, Kyoto, Japan).

### Bacterial Community Analysis

#### DNA Extraction and 16S rRNA Gene V4 Amplification and Sequencing

Bacterial DNA was extracted from tongue samples using MoBio PowerSoil (Qiagen) protocol with 0.7 mm garnet beads, as recommended by the manufacturer. The V3-V4 region of the bacterial genomic DNA was amplified using barcoded primers 515f (5′-GTGCCAGCMGCCGCGGTAA-3′) and 806r (5′-GGACTACHVGGGTWTCTAAT-3′). The barcoding PCR reaction contained the following: 2 uL 4 uM barcoded primer stock, 5 uL DNA, 2 uL Taq Buffer II (Invitrogen), 0.15 uL Taq enzyme (Invitrogen), and 10.85 uL PCR. The reactions are amplified in an Eppendorf mastercycler Thermocycler under the following conditions: initial denaturation step for 2 min at 95°C, followed by 30 cycles of 20 s denaturation at 95°C, 45 s of annealing at 50°C, and 90 s annealing at 72°C. A different barcode is used for each sample, allowing for pooling of samples for sequencing. All samples were pooled and sequenced on one lane of an Illumina HiSeq 2000 sequencer (Illumina, San Diego, CA) at the Baylor College of Medicine Human Genome Sequencing Center.

#### 16S rRNA Gene Data Analysis

Reads 1 and 2 from the Illumina-sequenced amplicons were de-multiplexed and imported as paired reads into CLC Genomics Workbench v10 (Qiagen), trimming adaptors and barcodes. Reads were merged using alignment parameters with a mismatch score of 2, a minimum total score of 8, and no tolerance of unaligned ends, and reads that failed to merge were discarded. Final reads were between 250 and 480 bp in length. Comparative quality of samples was assessed with the “filter samples” function, requiring all samples in the study to fall within 50% of the median number of reads to pass. All subject samples in the study passed the filter, with total reads per sample between 28,000 and 58,000. Sequences were clustered into OTUs against the HOMD 16s rRNA RefSeq database at 98% identity (version 14.5) (Chen et al., 2010). OTU clustering was set to allow creation of novel OTUs that failed to meet the similarity percentage required to match the selected database. The minimal occurrence to be defined as an OTU was set to 2. Novel OTUs were assigned identity by BLAST comparison to the bacterial 16s rRNA database at NCBI. Chimera detection was performed as part of OTU clustering with a chimera crossover cost of 3 using a Kmer size of 6. OTUs identified as chimeras were removed from the final OTU abundance table. Metadata was added to the final OTU abundance table to allow aggregation of data by time point, gender, subject, race, or oral hygiene frequency. Bar or pie charts were constructed to visualize the taxa present in each sample and across sample groups.

#### Calculation of Alpha and Beta Community Diversity and Significant Differences

Community diversity was assessed using the Microbial Genomics Diversity module of CLC Genomics Workbench. OTUs from the abundance table were aligned using MUSCLE with a required minimum abundance of 10. Aligned OTUs were used to construct a phylogenetic tree using Maximum Likelihood Phylogeny using the Neighbor Joining method and the Jukes Cantor substitution model. Alpha diversity measures were calculated for total OTUs, and Shannon's Entropy. Rarefication analysis was done by sub-sampling the OTU abundances in the different samples at a range of depths from 1 to 100,000; the number of different depths sampled was 20, with 100 replicates at each depth. Statistical significance in alpha diversity between cohorts and time points was calculated with one-way ANOVA and *post-hoc* tests by Bonferroni. PERMANOVA Analysis (Permutational Multivariate Analysis Of Variance) was used to detect significant differences in Beta diversity between groups. Differential abundance tests (non-parametric ANOVA) on the OTU frequency table were used to identify significant changes in the relative abundances of individual OTUs between groups. Differential abundance analysis values were calculated for: the max group means (maximum of the average RPKM's), –log2 fold change, fold change, standard *p*-value (significance at <0.05), and FDR *p*-value (false discovery rate corrected *p*-value).

#### Community Gene Content Using PICRUSt And LEFSE

Sequences were clustered into OTUs against the Greengenes database at 97% homology, and the minimal occurrence to be defined as an OTU was set to 2. OTU's that failed to match the database were discarded. Data tables were clustered by tongue cleaning frequency and time point, or dominant genus and time point, and relative abundance tables were imported into PICRUSt for estimation of community gene content (http://huttenhower.sph.harvard.edu/galaxy) using the Kyoto Encyclopedia of Genes and Genomes (KEGG) (Ogata et al., 1999). Significant enrichment of enzyme functions was identified using LEFSE (Segata et al., 2011).

### Quantitative RT-PCR for Copper-Containing Nitrite Reductase (EC 1.7.2.1)

DNA sequences for the nitric oxide-forming nitrite reductase gene [KEGG orthology K00368; (Cantera and Stein, 2007)] were downloaded from 18 strains of *H. parainfluenza*, 1 strain of *H. pittmaniae*, 2 strains of *H. parahaemolyticus*, and 9 commensal strains of *Neisseria (N. mucosa/sicca/subflava/weaveri/cinerea)*. The genes were aligned with the “create alignment” module of CLC Genomics, with a gap open cost of 10 and a gap extension cost of 1.0. The resulting alignment is shown in Supplemental Data Figure 8A, and using the “design primers” module, multiple pairs of universal degenerate primers were designed. These universal primers failed specificity testing by BLAST against whole bacterial genomes. A second round of genus specific universal primers were designed for the *Haemophilus* nitrite reductase gene *nirK*, and the *Neisseria* nitrite reductase gene *aniA*. These primers passed both BLAST specificity and PCR cross-testing against genomic DNA of *Haemophilus* oral taxon 851 strain # F0397 and *N. mucosa* strain C102 (BEI Resources, Manassas, VA) by melting curve analysis and agarose gel analysis. Other quality control testing for was performed as recommended for qRT-PCR by the manufacturer (Taylor et al., 2010). Universal PCR primers for bacterial 16s rDNA were 907R and 357F as previously described (Martin et al., 2002). All primers were synthesized by Sigma-Aldrich, The Woodlands, TX. Quantitative RT-PCR reactions were carried out using SsoAdvanced Universal SYBR Supermix (Bio-rad, Hercules, CA). Reactions were performed on a StepOnePlus Real-Time PCR System (ThermoFisher Scientific), in 20 μl reactions with 10 μl 2× Supermix, 1–5 μl template, and 300 nM of each primer. Standards were 10-fold serial dilutions of genomic DNA from *Haemophilus* oral taxon 851 strain # F0397 (for *Haemophilus* nitrite gene *nirK* PCR), or genomic DNA from *N. mucosa* (for *Neisserria aniA* nitrite gene and 16s rRNA PCR). Genome equivalents per ng of genomic DNA were calculated on a genome size of 2.3 mB for *Neisseria* and 2.0 mB for *Haemophilus*. Standards were run with triple technical replicates, unknowns and negative controls were run in duplicate. Each primer set was run on separate 96 well plates, and each primer set was run at least twice. The relative ratios of nitrite gene counts normalized to bacterial cell number were calculated as (*Haemophilus* gene counts + *Neisseria* gene counts) divided by total 16s gene counts.

## Results

### Blood Pressure Changes in Response to Treatment With and Recovery From Chlorhexidine

The subjects recruited for this study were all faculty, staff, or students at the University of Texas School of Dentistry at Houston. The study design consisted of a baseline visit, a visit after 7 days treatment with CHX, a 3-days recovery from treatment follow-up, and a 7-days follow-up (Supplemental Data Figure 1). Twenty-seven subjects completed the first stage of the study, and 26 subjects completed the entire study. There were 17 females and 10 males; the average age was 31.8 years, with the youngest subject 22 and the oldest 71 years of age (Table 1). The average resting blood pressure at baseline in this cohort was 113/78 mmHg, and both systolic and diastolic data points conformed to a normal distribution. Two-way repeated measures ANOVA of systolic blood pressure readings indicate that significant differences exist between time points (*p* = 0.017), and between individual subjects (*p* = 0.0001), with a significant interaction component (*p* = 0.005). *Post-hoc* analysis identified significant changes in systolic blood pressure between the treatment and 3-days recovery time points (115 vs. 111.5 mm Hg), as well as the 3-days recovery and 7-days recovery (111.5 vs. 113.3 mm Hg; Table 3). Diastolic blood pressure was also significantly different at these same points.

**Table 3:** Blood pressure values.

**SYSTOLIC VALUES BY TIMEPOINT**					
**Timepoint**	**Systolic mean**	**Minimum**	**Median**	**Maximum**	***N***
Baseline	113.0	99.0	113.0	129.0	27.0
Treatment CHX	115.0	91.7	114.0	135.7	27.0
Recovery 3 days	111.5	96.3	112.3	126.0	25.0
Recovery 7 days	113.3	94.7	113.7	132.0	26.0
**DIASTOLIC VALUES BY TIMEPOINT**					
**Timepoint**	**Diastolic mean**	**Minimum**	**Median**	**Maximum**	***N***
Baseline	78.1	69.3	77.7	89.7	27.0
Treatment CHX	78.4	65.0	78.3	95.7	27.0
Recovery 3 days	75.7	62.7	77.0	87.3	25.0
Recovery 7 days	79.0	68.3	78.5	96.0	26.0
**Statistical comparisons**	**Systolic blood pressure**		**Diastolic blood pressure**		
**Group vs. Group (Contrast)**	**Bonferroni** ***p*****-level**	**Fisher LSD** ***p*****-level**	**Bonferroni** ***p*****-level**	**Fisher LSD** ***p*****-level**	
Baseline vs. Treatment 7 days CHX	0.508	0.085	1.000	0.751	
Treatment 7 days CHX vs. Recovery 3 days	**0.014**	**0.002**	**0.025**	**0.004**	
Recovery 3 days vs. Recovery 7 days	0.136	**0.023**	**0.003**	**0.000**	
Baseline vs. Recovery 3 days	1.000	0.171	0.063	**0.010**	
Baseline vs. Recovery 7 days	1.000	0.344	1.000	0.306	
Treatment 7 days CHX vs. Recovery 7 days	1.000	0.444	1.000	0.477	

*Systolic and diastolic average values per time point are shown for all subjects in the study. Minimum, median, and maximum are also shown. N = number of data points per time point. Statistical comparisons for systolic and diastolic BP are also shown, including both Bonferroni and Fisher calculations. Significant p-values are shown in bold*.

### Individual Blood Pressure Responses to Treatment With Chlorhexidine

**Figure 2:**
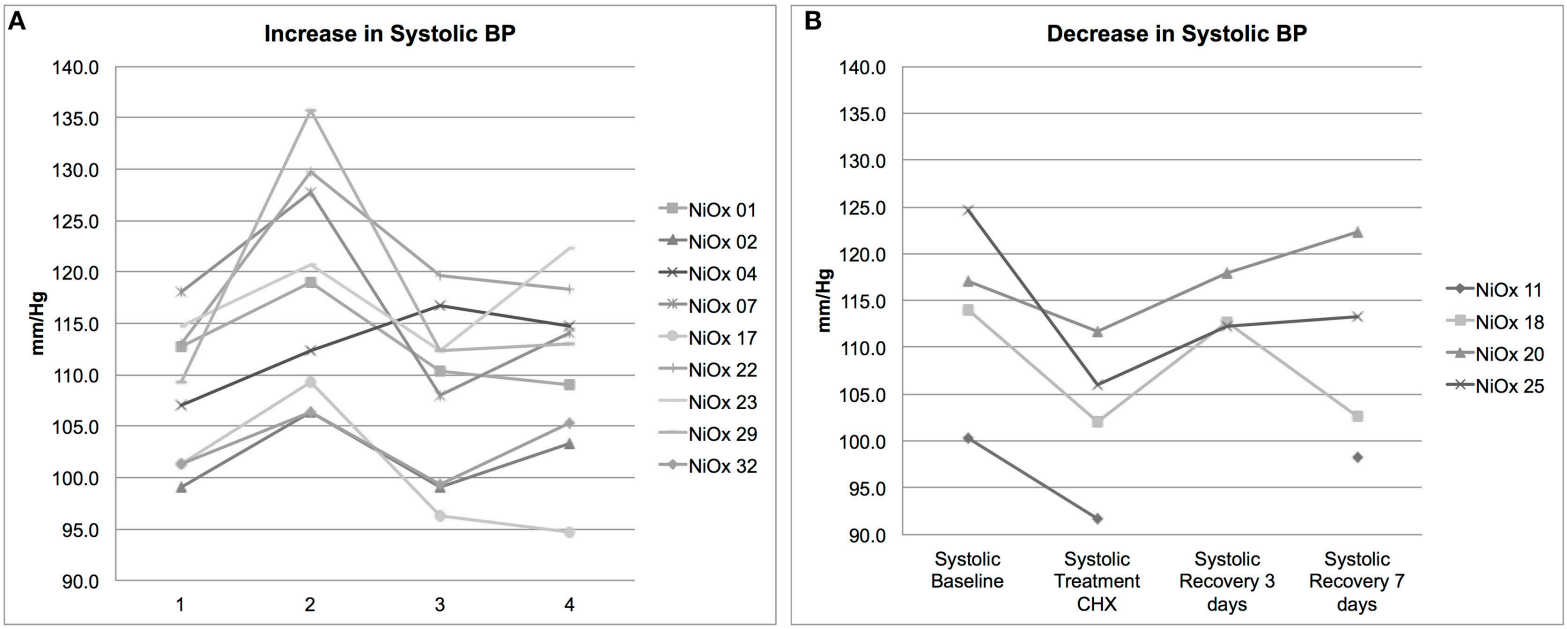
Individual systolic blood pressure responses. Individuals with a >5 mm/Hg change in blood pressure after CHX treatment are shown **(A)** Nine subjects had an increase in systolic BP. **(B)** Four subjects had a decrease in systolic BP. Each point represents an average of three systolic readings of resting BP. Subject NiOX 11 did not have BP readings taken at the third time point.

The time point-subject interaction p-levels indicate that there were significant individual differences in response to CHX. Assessment of systolic blood pressure results on within-subject data revealed that 13 subjects had changes in blood pressure >5 mm/Hg in response to CHX; 9 subjects had an increase in resting blood pressure after treatment with CHX, while four had a decrease (Figure 2). This bimodal response to CHX treatment was confirmed by comparison of the blood pressure distribution at baseline to the other time points by an F-test for Variances. Only the CHX treatment time point has significantly different variance compared to baseline (*p*-level two-tailed = 0.04). Thus, in our study, there is a significant difference in individual response after 7 days of treatment with CHX, and an overall significant decrease in systolic and diastolic blood pressure average in the cohort after recovery from CHX for 3 days. We next determined if any of our demographic or hygiene data could account for the difference in individual response after 7 days of treatment with CHX.

### Correlation Between Blood Pressure Response to CHX and Oral Hygiene Behaviors

At the first visit, subjects completed a demographic and oral hygiene survey. Subjects provided information on their age, gender, education level, number of times per day they brush their teeth, use dental floss, use mouthwash, clean their tongue, visit the dentist per year, and the types of oral care products used (Table 2). We utilized this data to investigate the different responses to treatment with CHX within our subject population. Both systolic and diastolic blood pressure was found to vary significantly by gender, with male subjects averaging 10 mm/Hg higher systolic and 5 mm/Hg higher diastolic than females. However, there was no significant interaction between gender and blood pressure response over the course of the study, indicating that there is no difference in response between males and females (Supplemental Data Figure 2). No significant variation was found between any other groups based on race/ethnicity, education, or other variables.

**Figure 3:**
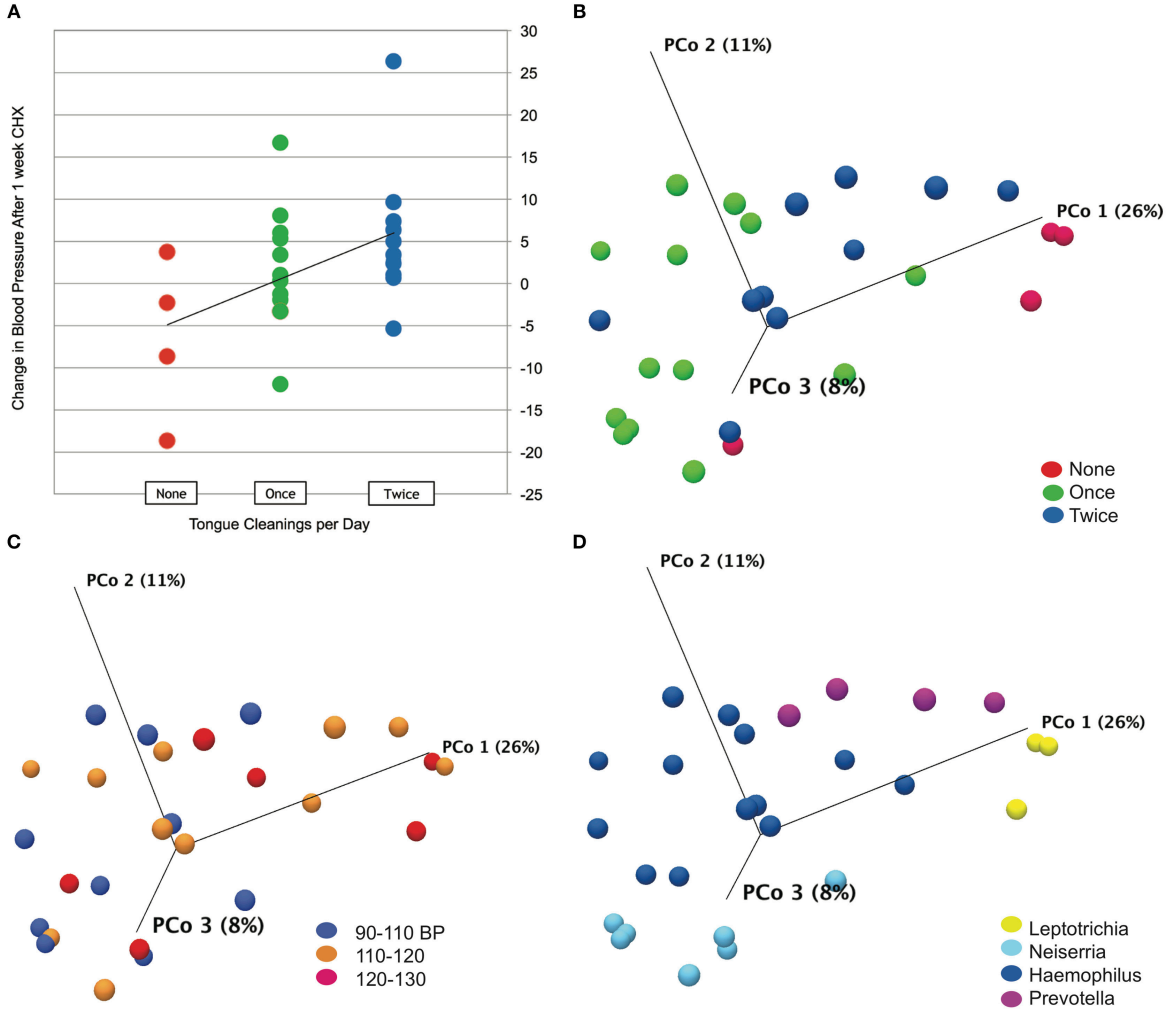
Tongue cleaning impact on blood pressure response and microbiome composition. **(A)** Correlation plot for blood pressure response to chlorhexidine and frequency of tongue hygiene. Spearmans's correlation coefficient was used to determine the strength of association. **(B)** Bray-Curtis Principle component plot of bacterial communities at baseline, colorized by tongue-cleaning frequency. **(C)** Bray-Curtis Principle component plot of bacterial communities at baseline, colorized by resting systolic blood pressure. **(D)** Bray-Curtis Principle component plot of bacterial communities at baseline, colorized by dominant bacterial genus.

Subject data, represented by change in systolic blood pressure between baseline and after the 1 week CHX treatment, was correlated with metadata categories using Spearman's correlation. No significant correlations were found for any demographic or oral hygiene data, except for tongue cleaning frequency. An R of 0.45 for correlation with tongue cleaning frequency was determined to be significant with a *p*-value of 0.03 (Figure 3A). Subjects who cleaned their tongue twice or more per day as part of their normal oral hygiene were more likely to have an increase in systolic blood pressure during use of CHX for 1 week. Subjects who did not clean their tongue on a daily basis were more likely to have a decrease in systolic blood pressure. Separating the subjects into cohorts based on tongue-cleaning frequency, we performed one-way ANOVA on blood pressure data by cohort, and confirmed the cohort of subjects who clean twice a day had a significant increase in systolic BP after 7 days CHX treatment, followed by a significant decrease at the 3-days recovery time point (Supplemental Data Figure 3, *p*-value = 0.003). The zero cleaning cohort shows the opposite response, with a significant decrease after 7 days CHX (117. 5 mm/Hg to 111.0 mm/Hg, *p*-value = 0.03), and a subsequent increase at the 3 days recovery (111.0–117.4). To confirm that the frequency of tongue cleaning was not tied to other demographic variables, we used the Chi Square test for gender, age-range and race-ethnicity, with no significant associations.

From these observations, we hypothesized that the frequency of tongue cleaning as a significant effector of blood pressure could result from a combination of possible effects. First, regular tongue cleaning may result in a baseline tongue microbiome that has a greater ability to reduce nitrate, and conversely, failure to clean the tongue daily may result in a microbiome composition that is unfavorable to nitrate reduction. Further, cleaning the tongue disrupts the papillary surface and could allow increased penetration of CHX, resulting in a greater community disruption in the two cleaning cohort. To investigate these mechanisms, we used 16S rDNA community analysis of tongue samples to elucidate the dynamics of the tongue microbiome in our subjects, to compare differences between time points and tongue hygiene cohorts.

### The Bacterial Microbiome of the Healthy Human Tongue

In tongue samples from 27 subjects at baseline, 272 unique OTUs were identified at the phylum level, representing eight different phyla of bacteria: Proteobacteria, Bacteroidetes, Firmicutes, Fusobacteria, Actinobacteria, Candidate division SR1, Spirochaetes, and Candidate division TM7. Members of the first five phyla were found in every subject (Supplemental Data Figure 4A), while the remaining phyla were found as a minority component in some subjects, resulting in combined abundance values <1%. This is consistent with other studies in adults, which demonstrated the same major phyla on the tongue (Jiang et al., 2012a; Mark Welch et al., 2014). In our study, Proteobacteria account for 40% of the OTUs identified across the cohort; Bacteroidetes account for 23%, Firmicutes for 19% (Table 4). The most common OTU in our cohort at baseline was *Haemophilus parainfluenza*, a commensal bacterium of the oral-pharyngeal flora and a member of the Proteobacteria. This organism accounted for 22% of the combined abundance in the cohort, followed by *Neisseria subflava* at 12%. The genus *Haemophilus* was the most numerous in 14 of the 27 subjects at baseline (Supplemental Data Figure 4B). Six subjects had *Neisseria* as the most common genus, while four subjects had *Prevotella* and three had *Leptotrichia*. Other bacteria identified in this study are shown in Supplemental Data Figure 4C, and all species-level identifications are based on OTU comparisons to the HOMD 16s rRNA RefSeq database at 98% identity (Chen et al., 2010).

**Table 4:** Average composition of the tongue microbiome across all subjects.

**Phylum: Genus**	**Average tongue microbiome composition by phylum**	**Average tongue microbiome composition by genus**	**High-low range**
Proteobacteria: Haemophilus	40%	25%	82–1%
Proteobacteria: Neisseria		13%	47–0.1%
Proteobacteria: Campylobacter		2%	9–0%
Bacteroidetes: Prevotella	23%	18%	46–1%
Bacteroidetes: Porphyromonas		3%	13–0%
Bacteroidetes: Flavobacteria		2%	3–0%
Firmicutes: Streptococcus	19%	9%	20–2%
Firmicutes: Veillonella		6%	15–1%
Firmicutes: Lachnospira		3%	18–0%
Firmicutes: Carnobacteria		1%	2–0%
Fusobacteria: Leptotrichia	12%	6%	34–0%
Fusobacteria: Fusobacteria		6%	19–0%
Actinobacteria: Actinomyces	5%	4%	13–0%
Actinobacteria: Micrococcus		1%	4–0%

*The first and second column lists the distribution of bacteria by phylum, in order of most common to least common. The third column shows the average composition by genus, and the last column indicates the highest percentage and lowest percentage for individuals in the study*.

### Effects of Tongue Hygiene and Demographics on Bacterial Community Composition

To test the effect of tongue cleaning behavior on microbiome composition, we generated Principle Component Plots for all samples at baseline. By PERMANOVA analysis, bacterial community distribution was significantly influenced by tongue cleaning frequency, with a Bray-Curtis ANOVA *p*-value of 0.00001, and Bonferroni *post-hoc* analysis indicated that all tongue-cleaning subgroups were significantly different from each other (Figure 3B). PERMANOVA values for systolic blood pressure (*p* = 0.009) and dominant genus (*p* = 0.00001) were also significant (Figures 3C,D). For systolic blood pressure, Bonferroni *post-hoc* analysis was significant only between groups 90–110 and 120–130 (*p* = 0.006). To further assess the demographic and behavioral influences on community composition, we used Chi Square to assess their correlations with the dominant bacterial genus per subject (Supplemental Data Figure 5). Frequency of tongue cleaning was found to have a significant correlation with the dominant bacterial genus on the tongue.

We next analyzed differences between hygiene cohorts at baseline with differential abundance analysis (Supplemental Data Table 1). The largest differences in composition were found between the zero cleanings cohort and the other two. There are significant differences in 33 different OTUs for subjects with zero cleanings/day compared to the other two groups, and only four OTUs different between the one and two-cleaning groups. Notably, the levels of *Leptotrichia sp*. are significantly higher in the zero-cleanings group, and the levels of two *Haemophilus* OTUs are significantly lower. Other OTUs lower in the zero-cleanings group are associated with *Veillonella parvula, Rothia muciliginosa*, and *Granulicatella adiacens*. Therefore, tongue-cleaning does have a significant impact on tongue bacterial community composition, and daily cleaning (either once or twice) results in an increased proportion of *H. parainfluenza* and other Proteobacteria, which are major nitrate and nitrite reducing species of the oral cavity (Hyde et al., 2014).

### Bacterial Community Responses to CHX Treatment

**Figure 4:**
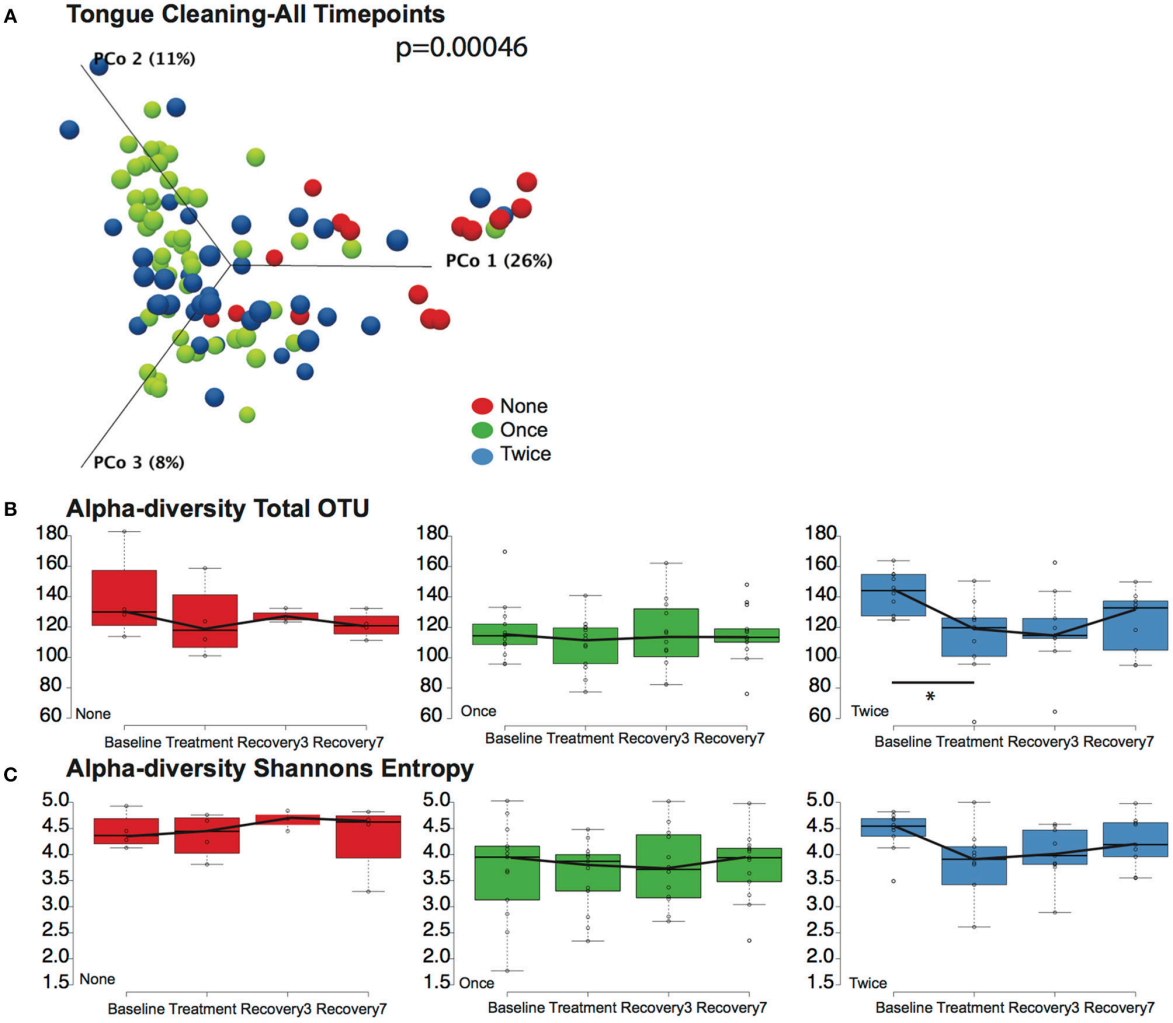
Effects of tongue hygiene and chlorhexidine exposure on community diversity and composition. **(A)** Bray-Curtis Principle component plot of bacterial communities across all time points, colorized by tongue cleaning frequency. The distribution between cohorts remains distinct; the PERMANOVA *p*-value is 0.00046. **(B)** Community richness as measured by Total OTUs. Only the two-cleaning per day cohort, shown in blue, had a significant change in richness after CHX treatment as noted by the asterisk. **(C)** Community diversity as measured by Shannon entropy. No significant differences were noted for any cohort.

We next tested the hypothesis that cleaning the tongue twice a day could influence blood pressure by causing a greater community disruption by CHX compared to the other cohorts. PERMANOVA analysis and Principle Component Plots for all time points demonstrated that samples continue to cluster by tongue-cleaning frequency (Figure 4A) after treatment with CHX. Further, there was no significant change in overall sample distribution for any tongue-cleaning cohort in response to CHX, based on PERMANOVA analysis (Supplemental Data Figure 6).

We further considered the impact of CHX exposure on community richness and diversity using Observed OTUs and Shannon Entropy calculations. Richness decreased upon CHX treatment for all three tongue-cleaning cohorts, but the only statistically significant decrease was in the two-cleaning group, between baseline and treatment with CHX (Figure 4B). Species diversity was also impacted in the two cleaning group after CHX exposure as assessed by Shannon Entropy, however this decrease was not statistically significant (Figure 4C).

Differential abundance analysis identified significant changes in some species in response to treatment with CHX (Supplemental Data Table 2). In the zero cleaning cohort three species had significant changes in abundance, while the one cleaning cohort had 10 and the two cleaning had 12. Of the species that increase in abundance after treatment, six are from the genus *Capnocytophaga*, which are known to be more resistant to CHX than other oral microbes (Wade and Addy, 1989). Collectively, this data demonstrates that twice-daily tongue cleaning in combination with CHX treatment has an impact on tongue microbiome richness, and a larger magnitude of impact relative to the other tongue-cleaning cohorts. The primary effect of tongue cleaning in general, however, appears to be on selection of a baseline community of bacteria enriched in nitrate-reducing species.

### Microbiome Viability and Metabolism After CHX Treatment

**Figure 5:**
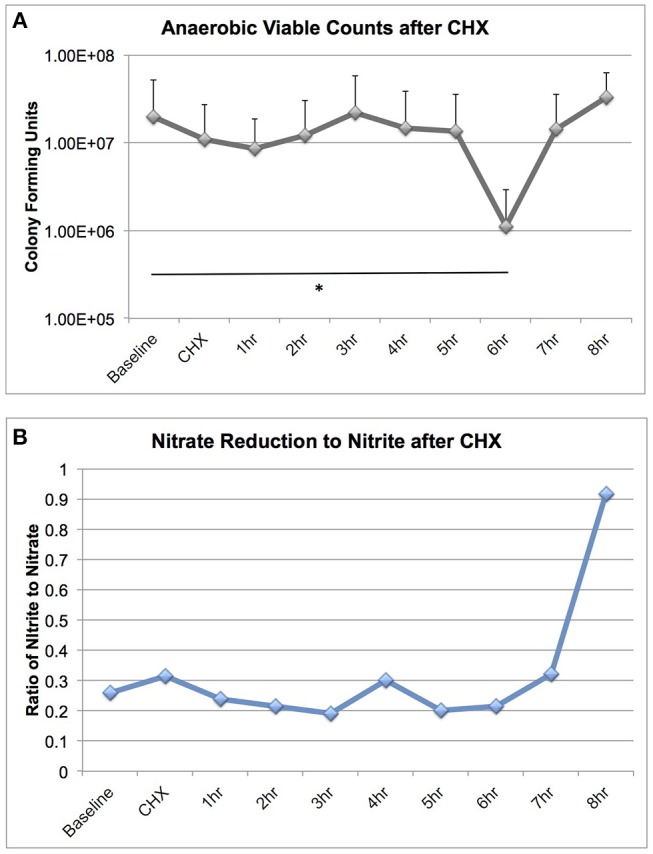
Bacterial viable counts and nitrate reductase activity recovered from tongue scrapings after CHX exposure. Six subjects collected tongue scraping samples over an 8 h time course after a 30 s rinse with 2% Peridex. Tongue samples were adjusted to a standardized starting OD600 and: **(A)** Serially diluted and plated for enumeration of viable counts on blood agar plates incubated anaerobically for 7 days and **(B)** Assessed for nitrate and nitrite values after anaerobic overnight incubation in 5 mM nitrate broth. Nitrate and nitrite results are shown as a ratio. Significant differences in viable counts between time points is indicated by an asterisk.

We were surprised to discover that CHX treatment did not cause large-scale changes in microbiome community structure, as it is a potent antimicrobial. We next assessed the effect of CHX exposure on tongue microbiome viability and nitrate reductase activity for 8 h after a 30 s rinse, on six subjects (Figure 5). CHX caused a significant reduction in bacterial viability, but this effect was only a 10-fold reduction, detectable 6 h after treatment. The dynamics of recovery from CHX exposure are notable, in that there was a rapid recovery in viable counts between 6 and 8 h, which corresponded with a significant increase in nitrate reduction to nitrite in the tongue samples. Thus, CHX mouthwash does not eradicate viable bacteria on the tongue, but instead causes a transient loss of viable counts, and the recovery from CHX treatment is associated with increased bacterial metabolic activity.

### Blood Pressure and CHX Responses by Dominant Genus Cohorts

**Figure 6:**
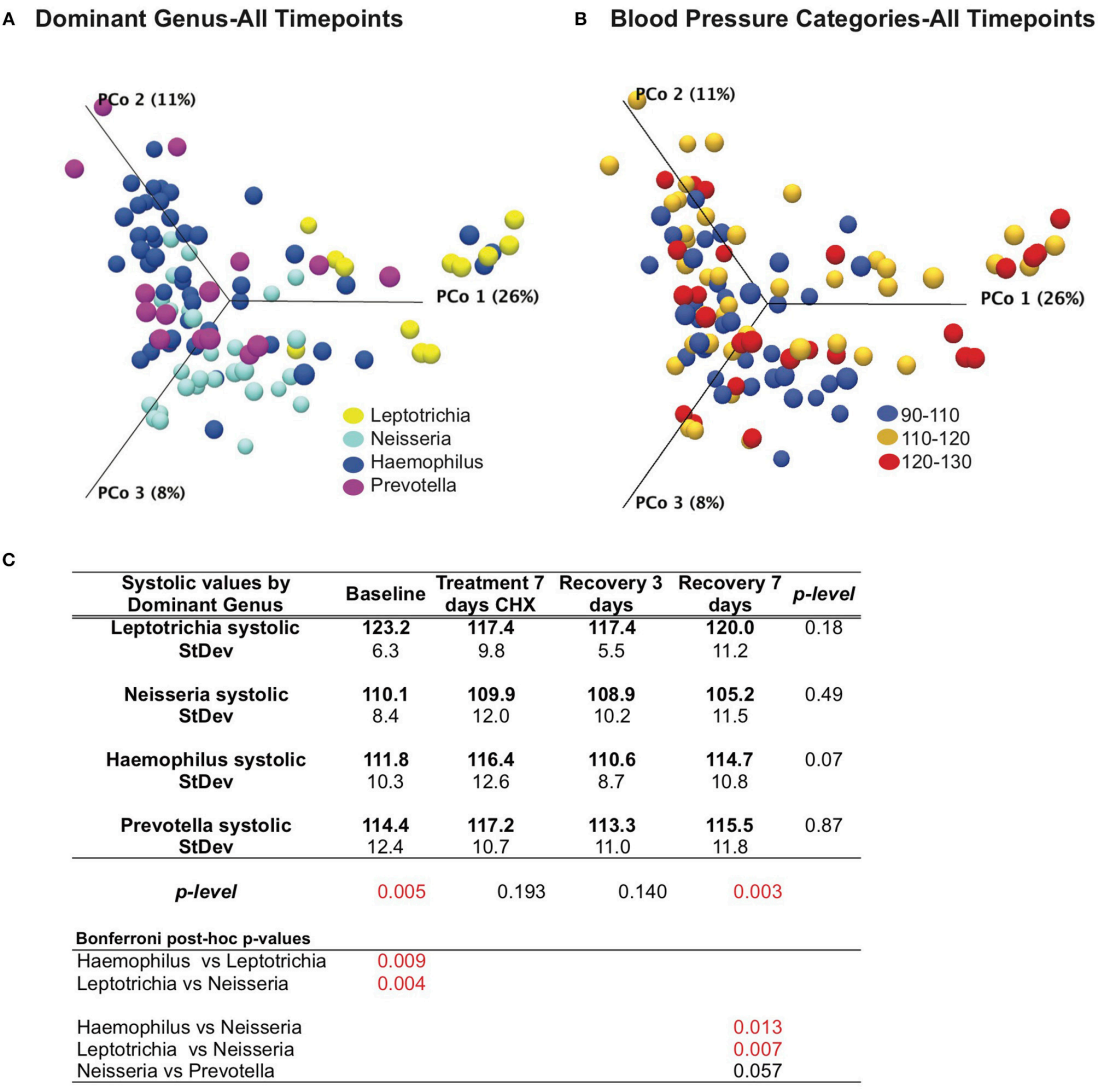
Effects of bacterial community and chlorhexidine exposure on systolic blood pressure. **(A)** Bray-Curtis Principle component plot of bacterial communities across all time points, colorized by dominant bacterial genus. **(B)** Bray-Curtis Principle component plot of bacterial communities across all time points, colorized by resting systolic blood pressure. **(C)** Statistical comparisons of systolic blood pressure between subjects, grouped by dominant bacterial genus.

We next revisited our blood pressure data and community responses to CHX from the perspective of the bacterial community type, and noted that bacterial communities associated with the lowest systolic blood pressure (90–110 mM/Hg) were localized to the left side of the principle component plot, and superimposed more closely with the *Haemophilus* and *Neisseria* groups (Figures 6A,B). In support of this observation, we found that there were significant differences (*p* = 0.005) between bacterial genus groups in systolic blood pressure at baseline (Figure 6C). The three subjects with *Leptotrichia* tongue communities have an average systolic blood pressure at baseline of 123.2 mm/Hg, while the *Neisseria* defined-cohort has a systolic average of 110 mm/Hg. *Post-hoc* analysis identified the *Leptotrichia* cohort as having significantly higher systolic BP than *Haemophilus* and *Neisseria* groups. No groups had significant changes in BP over the time course, although the *Haemophilus* group had a five point increase in BP after CHX use.

### Predicted Gene Content of the Human Tongue Microbiome

The PICRUSt program predicts gene family abundance (e.g., the metagenome) in microbial DNA samples for which only 16S rRNA gene data are available (Segata et al., 2011). Using PICRUSt, metagenomes were calculated for each tongue cleaning and bacterial genus cohort, and significant differences in relative abundance for gene families detected with linear discriminant analysis (LEfSe) with LDA score > 2 being considered significant. Across all samples, PICRUSt identified 4,295 genes with assigned KEGG orthology identifiers (KO). Comparing cohorts, 173 genes were found to be significantly different between tongue cleaning groups (Supplemental Data Table 3), and 674 genes between bacterial genus groups (Supplemental Data Table 4).

**Figure 7:**
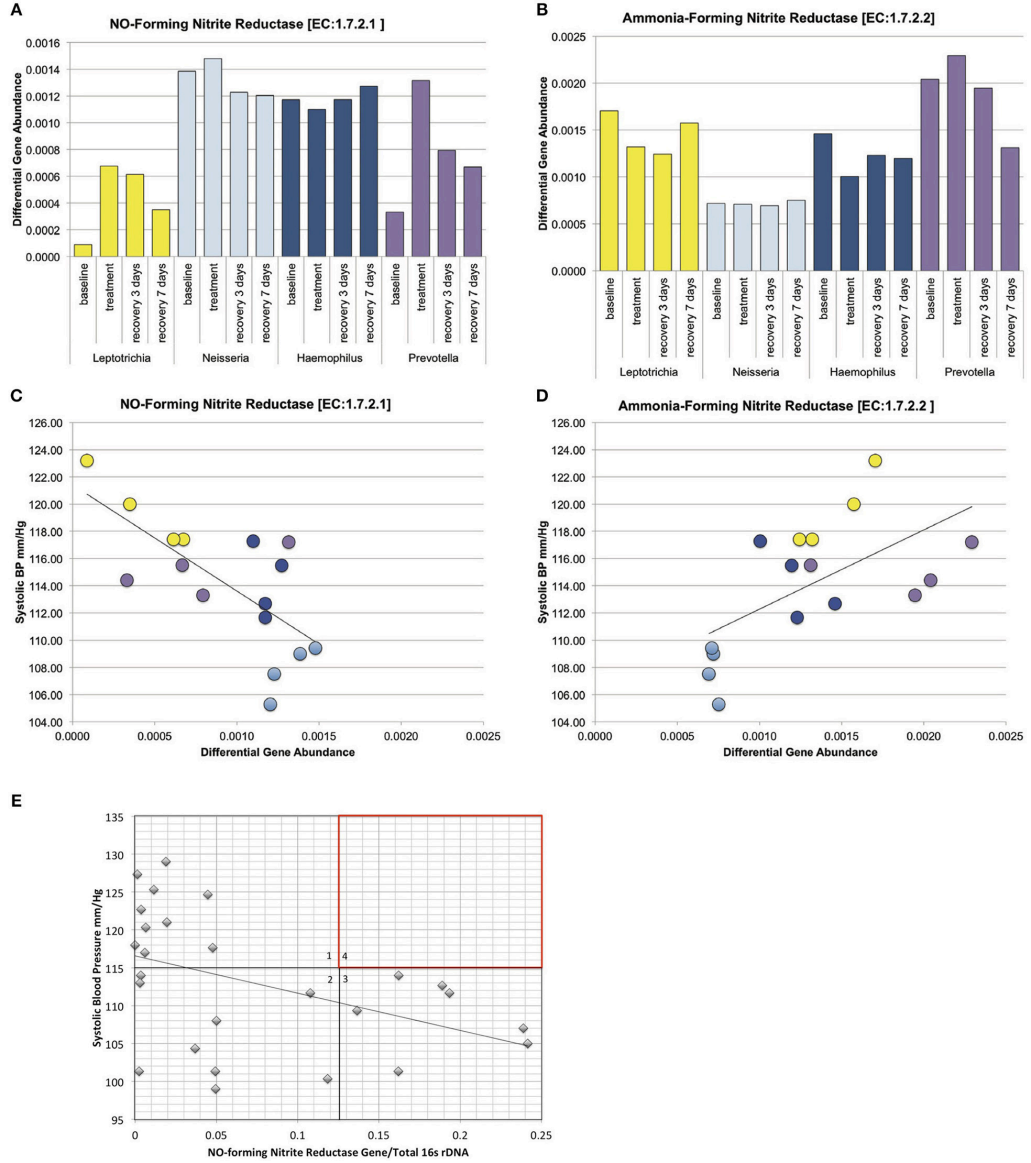
Relative gene abundance in the dominant genus cohorts. Subjects were grouped into cohorts based on microbiome type, named by the dominant genus found in the microbiome. **(A)** Predicted relative gene abundance for NO-forming nitrite reductase, which is significantly different between groups as determined by Lefse analysis. Predicted relative gene abundance was estimated by PICRUSt and represented as the percent of gene present in the community metagenome. **(B)** Predicted relative gene abundance for ammonia-forming nitrite reductase, which is significantly different between groups as determined by Lefse analysis. **(C)** NO-forming nitrite reductase gene abundance negatively correlated with changes in systolic blood pressure, with an *R* = −0.7 and *p* = 0.002. **(D)** Ammonia-producing nitrite reductase positively correlated with systolic blood pressure, with an *R* = 0.6 and *p* = 0.013. **(E)** The ratio of NO-forming nitrite reductase gene abundance in individual subjects at baseline, correlated with systolic blood pressure. The trend seen in the bacterial genus cohorts is reproduced in individual samples. The absence of subjects in quadrant 4 (red) implies that high ratios of NO-forming nitrite reductase on the oral microbiome may supplement host NO production and contribute to lower resting blood pressure.

Concerning the major bacterial enzymes associated with nitrate-nitrite metabolism (Figure 1A), NO-forming nitrite reductase [EC:1.7.2.1] varied significantly by both tongue-cleaning groups (Supplemental Data Figure 7B) and bacterial genus groups (Figure 7A); and [EC:1.7.2.2] ammonia-producing nitrite reductase was found to vary between the bacteria-genus cohorts (Figure 7B). Interestingly, the relative gene abundance for nitrate reductase did not significantly vary in either cohort (Supplemental Data Figure 7A). We confirmed the ability of all subjects to reduce nitrate to nitrite utilizing a 2-min *in vivo* oral rinse with 1 mM sodium nitrate. The average final concentration of nitrite for all subjects at baseline was 14 μM, and as predicted by the gene abundance, there was no significant difference in nitrite generation from nitrate between tongue-cleaning groups (Supplemental Data Figure 7C; *p* = 0.3) or bacterial-genus groups (data not shown).

We next performed correlation analysis across all cohorts at all time points for the NO-forming nitrite reductase gene. The gene did not correlate with blood pressure changes in the tongue cleaning cohort (Supplemental Data Figure 7D), but had a significant inverse correlation in the bacterial-genus cohorts (Figure 7C; *R* = −0.7; *p* = 0.0025). Additionally, the ammonia-producing nitrite reductase [EC:1.7.2.2] had a significant positive correlation with systolic BP (Figure 7D; *R* = 0.6; *p* = 0.013). Interestingly, although the *Neisseria* cohort and the *Haemophilus* cohort both have high levels of NO-forming nitrite reductase, the *Haemophilus* group has the higher amount of ammonia-producing reductase. Thus, high levels of NO-forming reductase are not sufficient, but rather high levels of NO-forming and low-levels of ammonia-forming produce the most favorable conditions for lower systolic BP.

### Quantitative PCR Detection of NO-Forming Nitrite Reductase in Individual Subjects

The correlation between increased NO-forming nitrite reductase and reduced systolic BP in the bacterial genus cohorts was further investigated in individual subject samples from the baseline time point. Primers specific for the NO-forming nitrite reductase of *Haemophilus* and *Neisseria* were designed to derive the Proteobacteria contribution to NO-forming gene content from the tongue bacterial DNA (Supplemental Data Figures 8A–C). Further, the total bacterial content of each sample was determined using quantitative RT-PCR primers for bacterial 16s rRNA, and the ratio of the total NO-forming gene to 16s rRNA in the sample was calculated (Figure 7E). We observed a significant inverse correlation of −0.44 (*p*-value = 0.02), further implicating a relationship between NO-forming bacterial nitrite reductase and resting systolic BP in individual subjects. We further note that not all subjects require a high concentration of NO forming nitrite reductase to achieve a healthy blood pressure. Five subjects in quadrant 2 have a NO-forming reductase/16s ratio of <0.05, but have a resting systolic BP of <110 mm/Hg. We predict that these subjects are producing sufficient endogenous NO, or have other BP regulation mechanisms in place that are bacterial NO-independent. In contrast, there are no subjects found in quadrant 4, implying that a high relative ratio of NO-forming nitrite reductase/16s ratio is contributing to a lower blood pressure.

## Discussion

This is the first longitudinal next-generation sequencing study demonstrating the impact of oral hygiene on the composition of the tongue microbiome. Tongue microbiome communities are of general interest in Eastern medicine because the appearance of the tongue coating is considered a manifestation of systemic health (Jiang et al., 2012a). In Western medicine interest has focused on the role of the microbiome in mucosal disorders (Docktor et al., 2012; de Paiva et al., 2016), and in dentistry the tongue microbiome has significant associations with halitosis (Ren et al., 2016). Regular tongue cleaning is recommended by the American Dental Association (http://www.mouthhealthy.org) based on evidence that cleaning can reduce the severity of halitosis (Pedrazzi et al., 2016), however there are no epidemiological data on tongue cleaning practices or frequency in the United States population. Based on this study, tongue cleaning assumes a new importance from the perspective of blood pressure regulation, as daily tongue cleaning appears to favor the increased abundance and metabolic activity of nitrate/nitrite metabolizing species, such as *H. parainfluenza* and commensal *Neisseria* spp. (Barth et al., 2009).

Consistent with the theory that the enterosalivary nitrate-nitrite-NO pathway is foundational to cardiovascular health, we demonstrated that our cohort of healthy subjects uniformly have tongue microbiomes encoding nitrate reductase activity. Interestingly, introduction of the antiseptic mouthwash CHX revealed different blood pressure responses, and a significant component of this response was determined to be the frequency of tongue cleaning. From our analysis, regular tongue cleaning results in a tongue microbiome that has a greater ability to reduce nitrite to NO, and conversely, failure to clean the tongue daily results in a microbiome composition that is less favorable to NO production, but instead appears to favor conversion of nitrite to ammonia. Introducing CHX to patients who clean their tongue twice a day results in a significant disruption of community alpha-diversity compared to the other groups, linking bacterial community disruption to changes in blood pressure. Our study also demonstrates a chemostat-like activity of the tongue microbiome, in that loss of population due to CHX exposure stimulates a rapid population recovery (Rai et al., 2019). Rapid recovery parallels an increase in nitrate reduction metabolic activity, and we propose that frequent tongue cleaning may activate bacterial metabolism and benefit the host through production of nitrite. Thus, regular tongue hygiene both selects for a favorable microbiome and “revs up” the activity of the community.

Although we did not survey host diet as a variable in our study, the composition of the human diet clearly has an impact on the composition of the microbiome, both in the gut and in the oral cavity. In a recent study on the salivary microbiome comparing vegans and omnivores (Hansen et al., 2018), the authors determine that the ratio of *Neisseria* to *Prevotella* is significantly higher in vegans. Further emphasizing the importance of dietary nitrate on the oral microbiome, Velmurugan et al. established in a randomized, double-blind study that sustained intake of nitrate-rich beetroot juice resulted in improved vascular function and also resulted in a significant increase in the percentage of *Neisseria flavscens* in the oral microbiome (Velmurugan et al., 2015). Both of these studies highlight the increase of commensal *Neisseria* spp. as a result of a diet high in nitrate-rich vegetables. Commensal Neisseria are part of the core oral microbiome in humans (Zaura et al., 2009), and reduction of nitrite to NO is obligatory for commensal Neisseria to survive in anaerobic conditions, such as those found in the crypts of the tongue (Bennett et al., 2014; Liu et al., 2015).

The data shown here provide static snapshots of what is undoubtedly a dynamic process. In our orally healthy cohort, the presence of nitrate reductase does not appear to be a rate-limiting component of the enterosalivary circulation of nitrate and subsequent reduction to nitrite. However, our experimental design has revealed a potential new aspect of enterosalivary circulation via the formation of NO in the oral cavity, in preference to formation of ammonia as the end product of nitrite. In subjects lacking other systemic health problems, the nitrite-reducing genetic content of the oral flora of the tongue significantly correlates with resting blood pressure values, with the putative mechanism through a supplemental contribution of nitrate reductase, and also nitric-oxide generating nitrite reductase. High levels of ammonia-forming nitrite reductase correlate with high blood pressure, and may result from “stealing” nitrite away from the NO pathway.

Although we have not established the mechanism by which generation of NO gas in the oral cavity could affect NO based vasodilation in the periphery, it is now well-established that NO generated in one biological compartment can affect NO homeostasis in distal tissue suggesting an endocrine function of NO (Elrod et al., 2008). Moreover, it is known that inhaled NO has systemic effects and can protect from myocardial ischemia reperfusion injury (Nagasaka et al., 2008) that is likely not due to NO itself but rather specific NO metabolites, nitrite and/or S-nitrosoglutatione (GSNO). Alternatively, NO gas produced in the lingual crypts could diffuse directly into the circulation through the highly vascularized tissue of the tongue.

Regulation of blood pressure is complex with multiple variables. There is a strong host component, including efficacy of endogenous NO production, weight, diet, gender, race, stress, and frequency of exercise (Rosendorff, 2013). We propose that the composition and metabolic activity of the oral microbiome should be considered an additional variable. Thirteen subjects on our study had changes of at least 5 mm/Hg in resting systolic blood pressure after CHX treatment, which is comparable to changes induced by manipulation of dietary salt intake (Graudal et al., 2017).

Manipulation of the human microbiome as a therapeutic target for disease management is on the near horizon. Screening the oral microbiome of resistant hypertensive patients may provide new insights into the etiology of their hypertension. The oral cavity is an attractive target for probiotic and/or prebiotic therapy because of the ease of access. The potential to restore the oral flora as a means to provide NO production is a completely new paradigm for NO biochemistry and physiology as well as to cardiovascular medicine and dentistry. These studies provide new insights into the host-oral microbiome symbiotic relationship. As NO is a ubiquitous signaling molecule, these systemic effects of these oral bacteria may have other significant effects on human health beyond maintenance of blood pressure.

## Data Availability

16S rRNA gene data from this study is deposited at the Sequence Read Archive (SRA) maintained at https://trace.ncbi.nlm.nih.gov/Traces/sra under bioproject title “The Oral Microbiome in Blood Pressure Regulation” 2444628.

## Ethics Statement

This study was carried out in accordance with the recommendations of the Committee for the Protection of Human Subjects at the University of Texas Health Science Center at Houston under study protocol number HSC-DB-14-0078. All subjects gave written informed consent in accordance with the Declaration of Helsinki.

## Author Contributions

GT, NA, and NB designed the study. NA, RW, B-YW, SE, IG, and KP recruited, screened, and sampled subjects. GT, D-HD, KR, NI, IS, and NB performed sample processing, nitrate-nitrite analysis, and quantitative RT-PCR. NJA and JP supervised bacterial DNA processing. GT performed bacterial community analysis. GT, NA, RW, B-YW, IS, EH, NJA, and NB wrote the manuscript.

### Conflict of Interest Statement

The authors of this manuscript declare the following competing interests: NB is a Founder and Shareholder of HumanN, a dietary supplement and functional nitric oxide nutrition company, a shareholder and consultant for SAJE Pharma, and he receives royalties on nitric oxide related patents from University of Texas. JP is President and Founder of Diversigen, a microbiome analysis service. NJA is Chief Scientific Officer at MicrobiomeDX, a microbiome analysis service. EH is Managing Editor at SynBioBeta. The remaining authors declare that the research was conducted in the absence of any commercial or financial relationships that could be construed as a potential conflict of interest.
