# Supplementary material for: Frequency of Tongue Cleaning Impacts the Human Tongue Microbiome Composition and Enterosalivary Circulation of Nitrate
Source: Front Cell Infect Microbiol. 2019 Mar 1;9:39. doi: 10.3389/fcimb.2019.00039 (PMC6406172; doi:10.3389/fcimb.2019.00039)
Supplement: Supplementary file 1 [file Data_Sheet_1.pdf]

Supplemental Data Figure 1

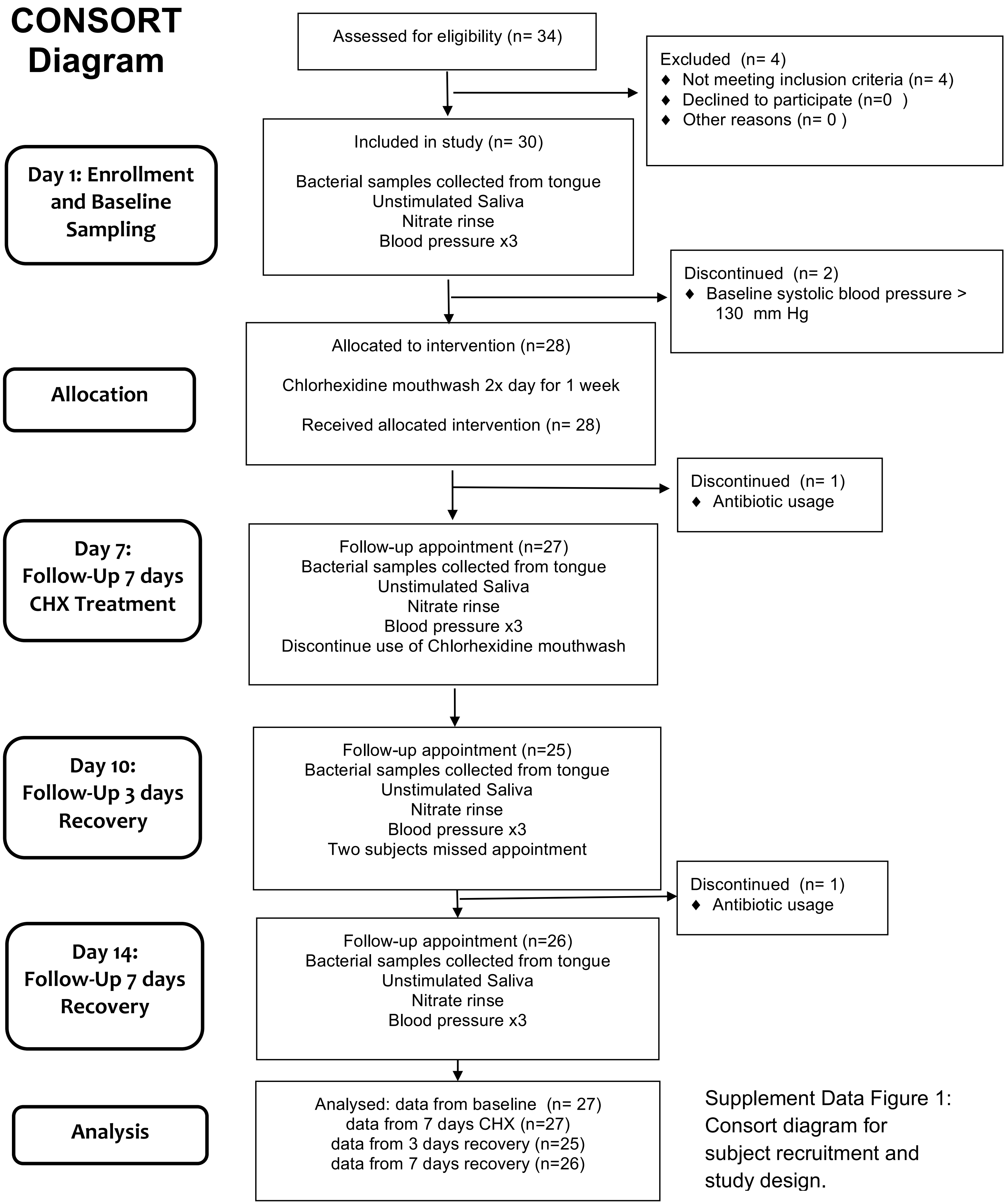

Supplement Data Figure 1: Consort diagram for subject recruitment and study design.

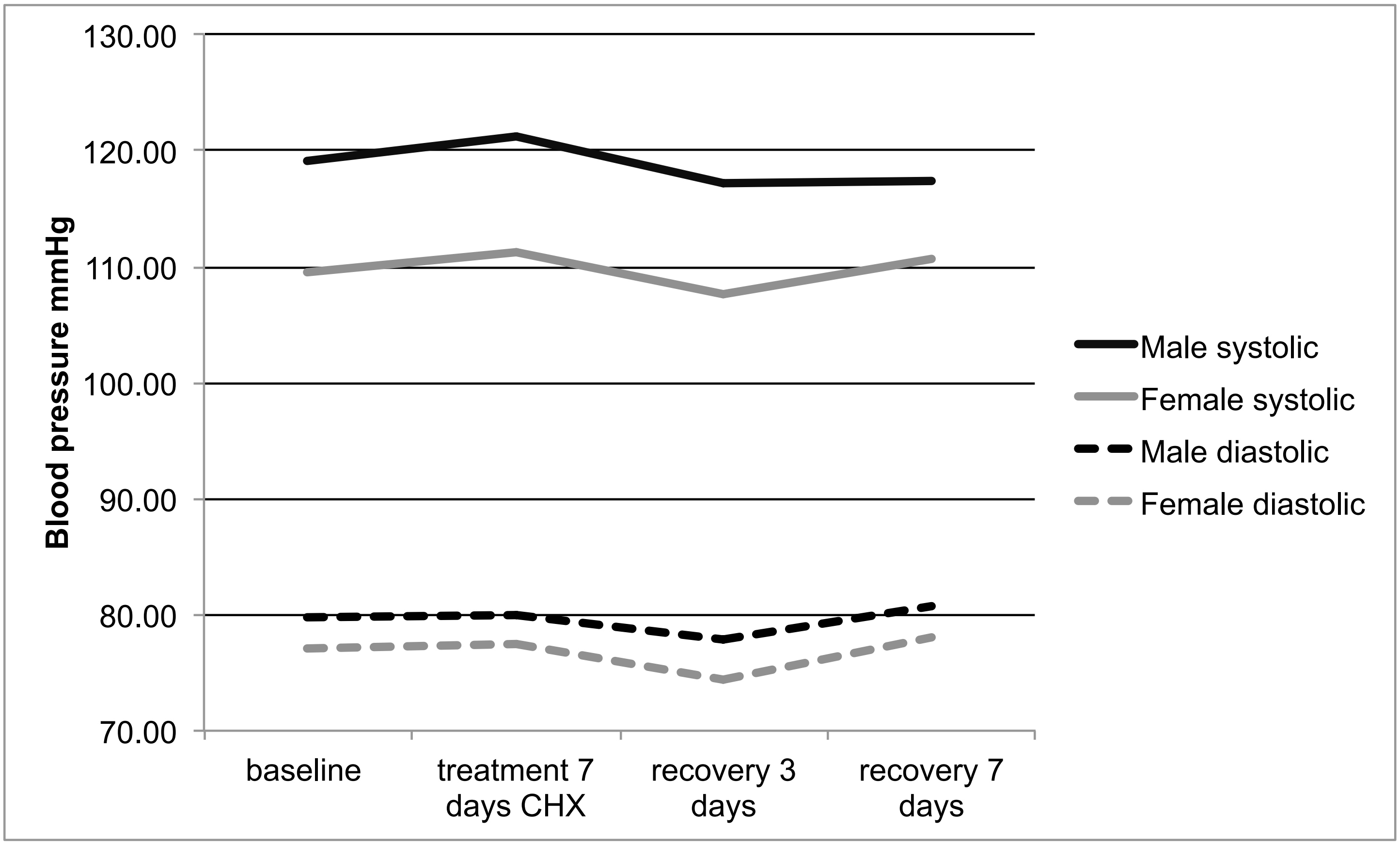

Supplemental Data Figure 2. Blood pressure response by gender. Blood pressure response by time point and gender is shown for systolic and diastolic readings. Statistical comparisons were made with two-way ANOVA, for gender and time point interaction; significant differences were found for gender (p=3.78E-5) but not in response to time point (p=0.59).

A

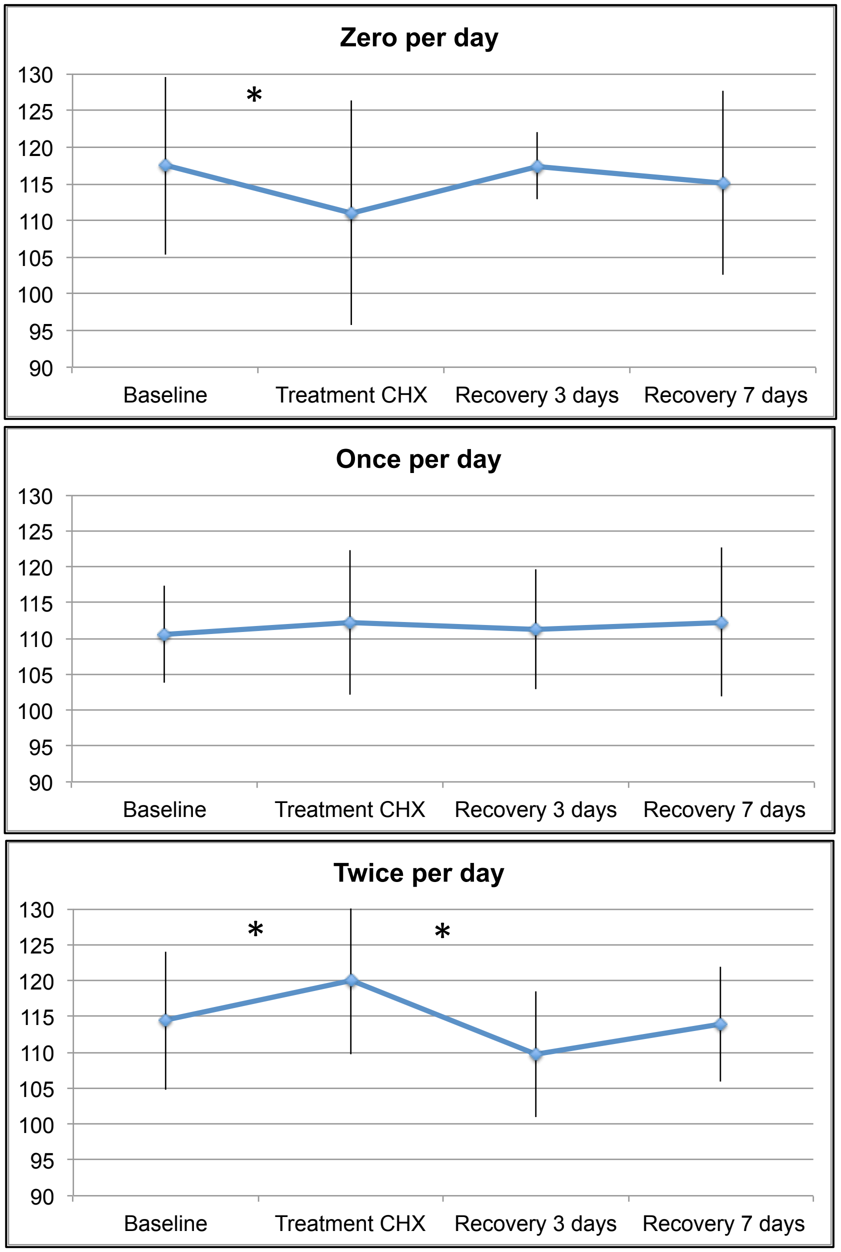

B

	**Baseline**	**Treatment 7 days CHX**	**Recovery 3 days**	**Recovery 7 days**	***p-level***
zero cleanings systolic	**117.5**	**111.0**	**117.4**	**114.1**	0.03
StDev	12.0	14.6	5.5	14.1	
one cleaning systolic	**111**	**112.3**	**111.3**	**112.3**	0.87
StDev	8.4	12.0	10.2	11.5	
two cleanings systolic	**114.4**	**120.0**	**109.7**	**114.0**	0.003
StDev	10.3	11.0	9.4	9.5	

Supplemental Data Figure 3. Blood pressure response by tongue cleaning frequency. a. Blood pressure response by time point and by tongue cleaning frequency is shown for systolic readings. $p < 0.05$= * **b.** One-way ANOVA p-values; both the zero and two cleanings cohorts have statistically significant changes in blood pressure in response to 7 days CHX exposure, as assessed by post-hoc analysis by Bonferroni.

A.

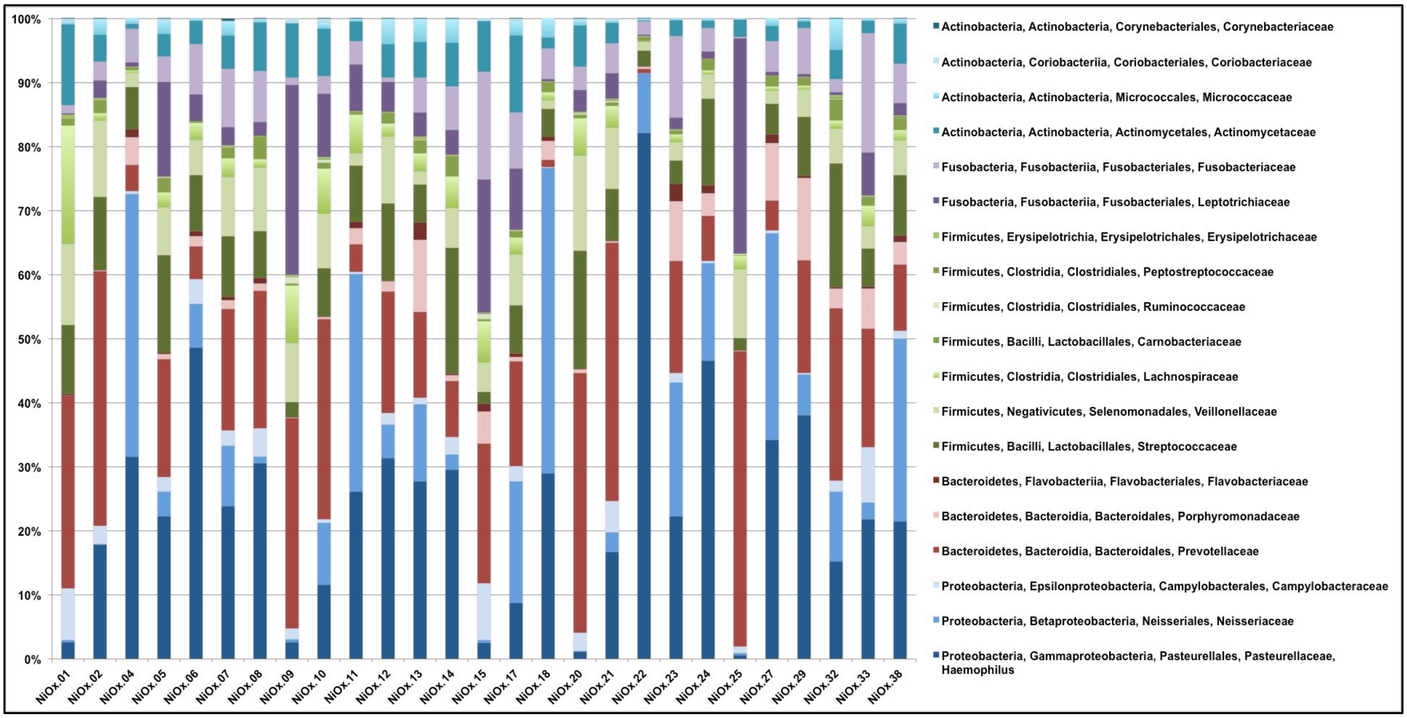

B.

Subject	Dominant Phyla	% Abundance	Dominant Genus	% Abundance
NiOx.01	Firmicutes	43%	*Prevotella*	28%
NiOx.02	Bacteroidetes	40%	*Prevotella*	36%
NiOx.04	Proteobacteria	72%	*Neiserria*	40%
NiOx.05	Proteobacteria	29%	*Haemophilus*	22%
NiOx.06	Proteobacteria	60%	*Haemophilus*	49%
NiOx.07	Proteobacteria	36%	*Haemophilus*	24%
NiOx.08	Proteobacteria	36%	*Haemophilus*	31%
NiOx.09	Bacteroidetes	33%	*Leptotrichia*	30%
NiOx.10	Bacteroidetes	31%	*Haemophilus*	28%
NiOx.11	Proteobacteria	60%	*Neiserria*	33%
NiOx.12	Proteobacteria	39%	*Haemophilus*	32%
NiOx.13	Proteobacteria	41%	*Haemophilus*	27%
NiOx.14	Firmicutes	36%	*Haemophilus*	29%
NiOx.15	Fusobacteria	37%	*Leptotrichia*	20%
NiOx.17	Proteobacteria	30%	*Neiserria*	19%
NiOx.18	Proteobacteria	77%	*Neiserria*	48%
NiOx.20	Bacteroidetes	41%	*Prevotella*	40%
NiOx.21	Bacteroidetes	41%	*Prevotella*	39%
NiOx.22	Proteobacteria	91%	*Haemophilus*	82%
NiOx.23	Proteobacteria	43%	*Neiserria*	20%
NiOx.24	Proteobacteria	62%	*Haemophilus*	47%
NiOx.25	Bacteroidetes	46%	*Leptotrichia*	34%
NiOx.27	Proteobacteria	66%	*Neiserria*	34%
NiOx.29	Proteobacteria	44%	*Haemophilus*	38%
NiOx.32	Firmicutes	30%	*Haemophilus*	24%
NiOx.33	Proteobacteria	33%	*Haemophilus*	22%
NiOx.38	Proteobacteria	50%	*Neiserria*	28%

C.

Proteobacteria	***Bacteroidetes***	***Firmicutes***
Campylobacter concisus	*Alloprevotella rava*	*Catonella morbi*
Campylobacter rectus	*Alloprevotella sp.*	*Gemella morbillorum*
Haemophilus influenzae	*Bergeyella sp.*	*Granulicatella adiacens*
Haemophilus parainfluenzae	*Capnocytophaga gingivalis*	*Lachnoanaerobaculum orale*
Haemophilus paraphrohaemolyticus	*Capnocytophaga granulosa*	*Lachnoanaerobaculum umeaense*
Haemophilus pittmaniae	*Capnocytophaga leadbetteri*	*Lachnospiraceae [G-2] sp.*
Haemophilus sp.	*Capnocytophaga sputigena*	*Megasphaera micronuciformis*
Kingella oralis	*Porphyromonas sp.*	*Moryella sp.*
Neisseria flava	*Prevotella aurantiaca*	*Oribacterium asaccharolyticum*
Neisseria sp.	***Prevotella histicola***	*Oribacterium sinus*
Neisseria subflava	***Prevotella melaninogenica***	*Rothia aeria*
	Prevotella nanceiensis	*Rothia mucilaginosa*
	Prevotella oulorum	*Rothia mucilaginosa*
	Prevotella pallens	*Ruminococcaceae [G-1] sp*
	Prevotella salivae	*Selenomonas sp.*
	Prevotella sp.	*Solobacterium moorei*
	Prevotella veroralis	*Stomatobaculum longum*
	Tannerella sp.	*Stomatobaculum sp.*
		Streptococcus cristatus
Fusobacterium	***Actinobacteria***	*Streptococcus vestibularis*
Fusobacterium periodonticum	*Actinomyces graevenitzii*	*Veillonella dispar*
Fusobacterium nucleatum subsp. animalis	*Actinomyces sp.*	*Veillonella parvula*
Fusobacterium nucleatum subsp. polymorphum	*Actinomyces lingnae*	*Veillonella rogosae*
Leptotrichia sp.	*Aggregatibacter sp.*	
Leptotrichia wadei	*Atopobium parvulum*	

Supplemental Data Figure 4. Tongue microbiome composition for each subject at baseline. a. The proportional composition of the tongue microbiome is diagramed, with each major phylum represented by a color family. Actinobacteria are represented in aqua, Fusobacteria in purple, Firmicutes in green, Bacteroidetes in red, and Proteobacteria in blue. **b.** The most common phlya and genus, per subject. **c.** Species found in at least two subjects, in at least 1% abundance, as identified by comparison to the HOMD 16s rRNA RefSeq database at 98% identity. Species found with the highest abundance are shown in bold.

A

Demographics Tongue Cleaning Cohorts

	**Zero**	**One**	**Two**
**Gender**	*Count*	*Count*	*Count*
Male	2	5	3
Female	2	8	7
**Age**	*Count*	*Count*	*Count*
20 To 30	3	7	6
30 To 40	-	4	2
40 To 50	1	2	1
50 and over	-	-	1
**Race and Ethnicity**	*Count*	*Count*	*Count*
African-American	-	-	3
Caucasian	3	7	4
Asian	-	4	1
Hispanic	1	2	-
Middle Eastern	-	-	1
Asian-Caucasian	-	-	1
**Tongue Microbiome Genus**	*Count*	*Count*	*Count*
Leptotrichia	3	-	-
Neisseria	1	5	1
Haemophilus	-	8	5
Prevotella	-	-	4

B

Demographics Dominant Genus Cohorts

	**Leptotrichia**	**Neisseria**	**Haemophilus**	**Prevotella**
**Gender**	*Count*	*Count*	*Count*	*Count*
Male	2	2	5	1
Female	1	5	8	3
**Age**	*Count*	*Count*	*Count*	*Count*
20 To 30	2	4	9	1
30 To 40	-	2	3	1
40 To 50	1	1	1	1
50 and over	-	-	-	1
**Race and Ethnicity**	*Count*	*Count*	*Count*	*Count*
African-American	-	-	2	1
Caucasian	3	2	8	1
Asian	-	3	2	-
Hispanic	-	2	1	-
Middle Eastern	-	-	-	1
Asian-Caucasian	-	-	-	1

C

Demographics Resting Blood Pressure at Baseline Cohorts

	**90-110**	**110-120**	**120-130**
**Gender**	*Count*	*Count*	*Count*
Male	1	4	5
Female	9	7	1
**Age**	*Count*	*Count*	*Count*
20 To 30	7	4	5
30 To 40	1	4	1
40 To 50	1	2	-
50 and over	1	1	-
**Race and Ethnicity**	*Count*	*Count*	*Count*
African-American	1	1	1
Caucasian	2	5	4
Asian	2	3	-
Hispanic	4	2	-
Middle Eastern	1	-	-
Asian-Caucasian	-	-	1
**Tongue Microbiome Genus**	*Count*	*Count*	*Count*
Leptotrichia	-	1	2
Neisseria	3	2	1
Haemophilus	5	6	2
Prevotella	2	2	1

D

Cross Tabulation and Chi-Square-Dominant Bacterial Genus vs Tongue Cleaning Frequency

Row Variable **# times clean tongue per day**

Column Variable **Dominant genus**

Observed Frequencies

Variable	*Haemophilus*	*Leptotrichia*	*Neisseria*	*Prevotella*	Total
Zero	0	3	1	0	4
Once	8	0	5	0	13
Twice	5	0	1	4	10
Total	13	3	7	4	27

Expected Frequencies

Variable	*Haemophilus*	*Leptotrichia*	*Neisseria*	*Prevotella*
Zero	1.93	0.44	1.04	0.59
Once	6.26	1.44	3.37	1.93
Twice	4.81	1.11	2.59	1.48

Chi-squared Values

Variable	*Haemophilus*	*Leptotrichia*	*Neisseria*	*Prevotella*
Zero	1.93	14.69	0.00	0.59
Once	0.48	1.44	0.79	1.93
Twice	0.01	1.11	0.98	4.28

Summary

Chi-square	2.823E+1
d.f.	6
p-level > X	8.486E-5
H0 (5%)	*rejected*

Supplemental Data Figure 5. Chi Square and Demographics. The demographic determinants for study participants arranged by tongue cleanings cohorts (**a**) dominant bacterial genus (**b**) or resting systolic blood pressure (**c**). Tongue cleaning frequency and dominant bacterial genus associations were tested with Chi square, and found to be significant (**d**).

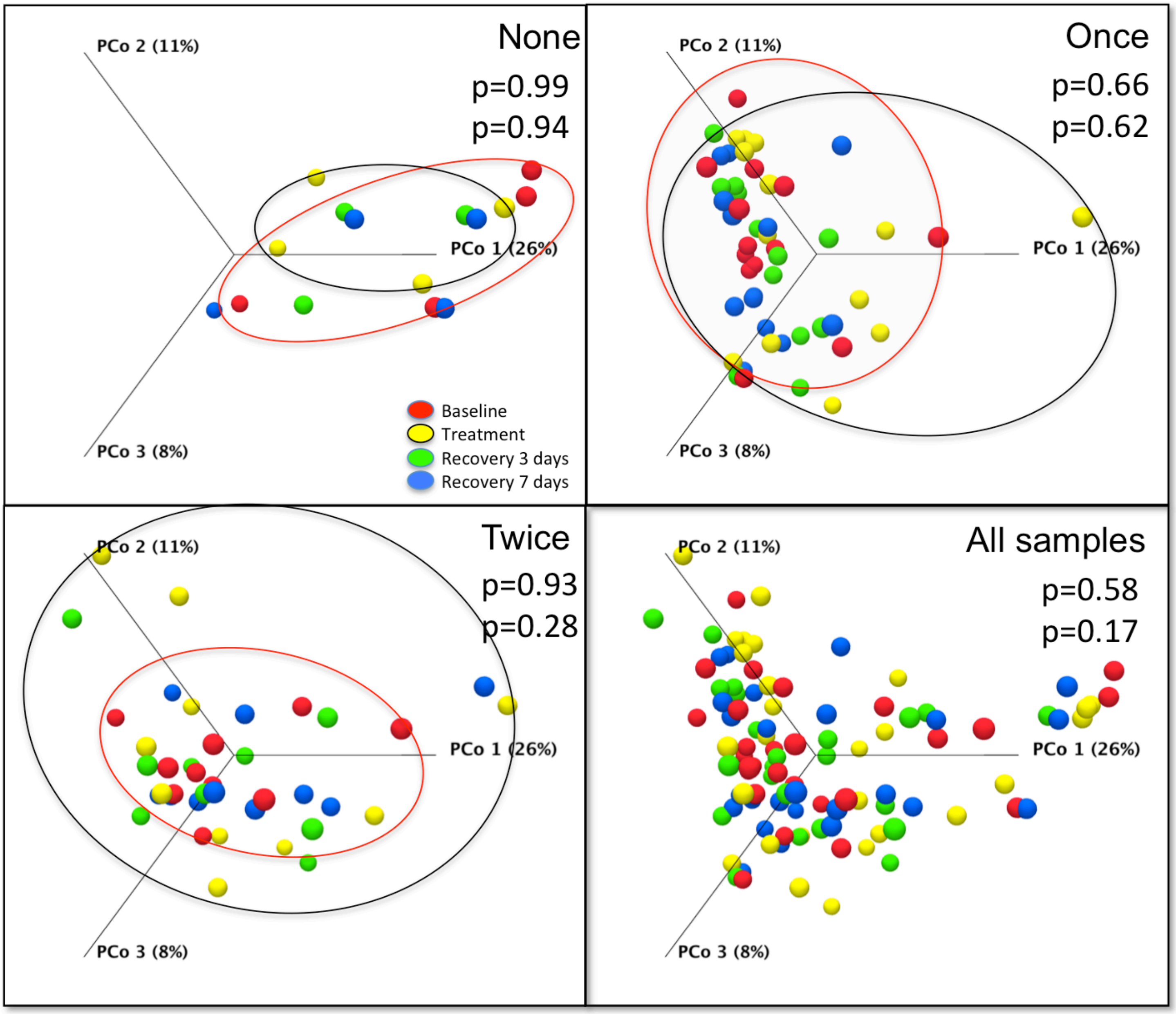

Supplemental Data Figure 6. Principle Component Analysis and PERMANOVA Results for Tongue cleaning cohorts over all time points. Baseline communities are circled in red, and communities after treatment with CHX are circled in black. PERMANOVA p values comparing sample distributions between all time points is shown in the upper right for each cohort panel, followed by the p value for comparison between baseline and treatment only.

Supplemental Data Figure 7

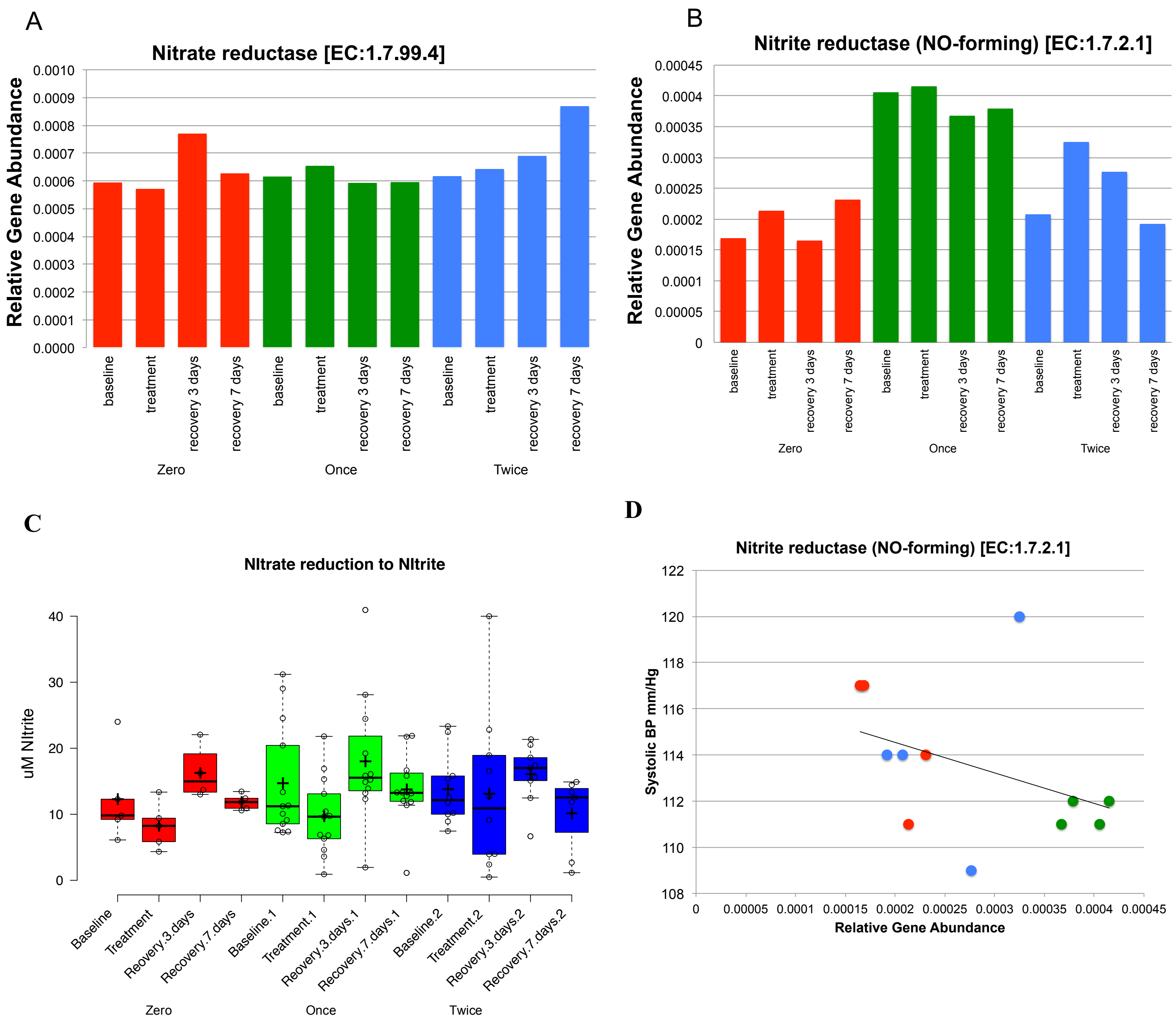

Supplemental Data Figure 7. Relative Gene Abundance and Nitrate Conversion to Nitrite in the Tongue Cleaning Cohorts.

a. Predicted relative gene abundance for nitrate reductase, as estimated by PICRUSt and represented as the percent of nitrate reductase gene present in the community metagenome. Nitrate reductase abundance was not significantly different between groups, as determined by Lefse analysis. **b.** Predicted relative gene abundance for NO-forming nitrite reductase, which is significantly different between groups. **c.** Study subjects rinsed for 2 minutes with a 1 mM solution of nitrate, and nitrate reductase activity inferred by measurement of resulting nitrite at the solution. Nitrite final concentration in micromoles is shown, with the mean as a black line and the median as a plus sign. No significant difference in nitrate reduction was found between any groups, by ANOVA. **d.** Nitrite reductase gene abundance varies significantly between tongue-cleaning groups, however does not significantly correlate with changes in systolic blood pressure, with an R= -.39 and p= 0.02.

A

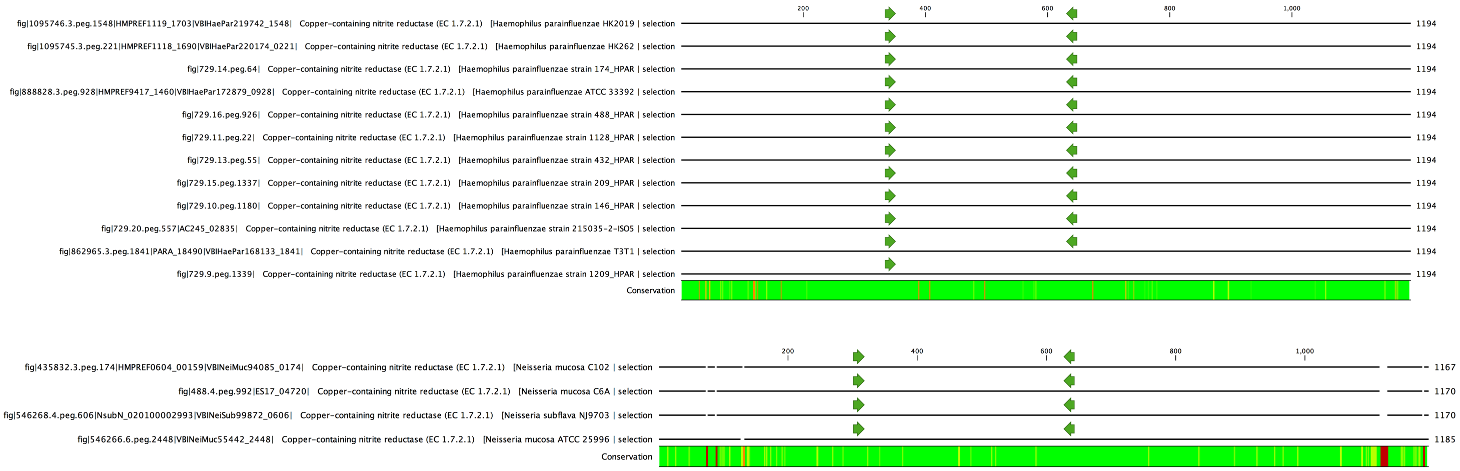

B

Primer name	Sequence	Product size bp	Reaction temperature Celcius	Annealing temperature Celcius
Haemophilus 129	CAAGCRGARGAAAARSCC	149	50	50
Haemophilus 275	TCAAGTGCGGTYAGTTTY			52
Neisseria 861	TCKTCYTTCCACGTWATCG	169	53	54
Neisseria 1012	GGAAGATRGAGTGGTCAA			52
Universal qPCR 907R	CCGTCAATTCMTTTRAGTTT	562-593	50	64
Universal qPCR 357F	CTCCTACGGGAGGCAGCAG			52

C

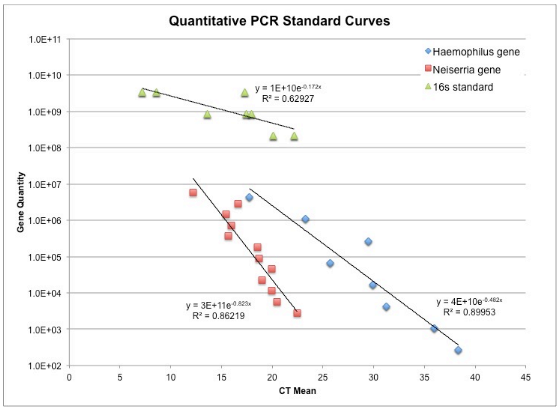

Supplemental Data Figure 8. Quantitative RT-PCR for NO-forming nitrite reductase gene. a, alignment of nitrite reductase genes from oral bacteria in the genus *Haemophilus* and *Neisseria*, with primer binding sites for genus specific primers as green arrows. Conservation at the DNA level is shown as a color chart below the alignment, with 0% conservation in black and 100% conservation in green. **b,** Primer sets for amplification of *Haemophilus* nitrite reductase, *Neisseria* nitrite reductase, or the bacterial 16s rRNA gene. **c,** Standard curves for each quantitative RT-PCR reaction.
